# The *Penicillium chrysogenum* transporter *Pc*AraT enables high-affinity, glucose-insensitive l-arabinose transport in *Saccharomyces cerevisiae*

**DOI:** 10.1186/s13068-018-1047-6

**Published:** 2018-03-13

**Authors:** Jasmine M. Bracher, Maarten D. Verhoeven, H. Wouter Wisselink, Barbara Crimi, Jeroen G. Nijland, Arnold J. M. Driessen, Paul Klaassen, Antonius J. A. van Maris, Jean-Marc G. Daran, Jack T. Pronk

**Affiliations:** 1 Department of Biotechnology, Delft University of Technology, Van der Maasweg 9, 2629 HZ Delft, The Netherlands; 2 Department of Molecular Microbiology, Groningen Biomolecular Sciences and Biotechnology Institute (GBB), University of Groningen, Nijenborgh 7, 9747 AG Groningen, The Netherlands; 3 DSM Biotechnology Centre, Alexander Fleminglaan 1, 2613 AX Delft, The Netherlands; 4 Present Address: Isobionics, Urmonderbaan 22-B 45, 6167 RD Geleen, The Netherlands; 5 Present Address: Institut de Génétique Humaine, UMR9002-CNRS-UM, 141 rue de la Cardonille, 34396 Montpellier, France; 6 Present Address: Division of Industrial Biotechnology, School of Biotechnology, KTH Royal Institute of Technology, AlbaNova University Centre, 20691 Stockholm, Sweden

**Keywords:** *Penicillium*, Transcriptome, Sugar transport, Proton symport, l-Arabinose transporter, Second-generation bioethanol, Yeast, Metabolic engineering

## Abstract

**Background:**

l-Arabinose occurs at economically relevant levels in lignocellulosic hydrolysates. Its low-affinity uptake via the *Saccharomyces cerevisiae* Gal2 galactose transporter is inhibited by d-glucose. Especially at low concentrations of l-arabinose, uptake is an important rate-controlling step in the complete conversion of these feedstocks by engineered pentose-metabolizing *S. cerevisiae* strains.

**Results:**

Chemostat-based transcriptome analysis yielded 16 putative sugar transporter genes in the filamentous fungus *Penicillium chrysogenum* whose transcript levels were at least threefold higher in l-arabinose-limited cultures than in d-glucose-limited and ethanol-limited cultures. Of five genes, that encoded putative transport proteins and showed an over 30-fold higher transcript level in l-arabinose-grown cultures compared to d-glucose-grown cultures, only one (Pc20g01790) restored growth on l-arabinose upon expression in an engineered l-arabinose-fermenting *S. cerevisiae* strain in which the endogenous l-arabinose transporter, *GAL2*, had been deleted. Sugar transport assays indicated that this fungal transporter, designated as *Pc*AraT, is a high-affinity (*K*_m_ = 0.13 mM), high-specificity l-arabinose-proton symporter that does not transport d-xylose or d-glucose. An l-arabinose-metabolizing *S. cerevisiae* strain in which *GAL2* was replaced by *PcaraT* showed 450-fold lower residual substrate concentrations in l-arabinose-limited chemostat cultures than a congenic strain in which l-arabinose import depended on Gal2 (4.2 × 10^−3^ and 1.8 g L^−1^, respectively). Inhibition of l-arabinose transport by the most abundant sugars in hydrolysates, d-glucose and d-xylose was far less pronounced than observed with Gal2. Expression of *Pc*AraT in a hexose-phosphorylation-deficient, l-arabinose-metabolizing *S. cerevisiae* strain enabled growth in media supplemented with both 20 g L^−1^ l-arabinose and 20 g L^−1^ d-glucose, which completely inhibited growth of a congenic strain in the same condition that depended on l-arabinose transport via Gal2.

**Conclusion:**

Its high affinity and specificity for l-arabinose, combined with limited sensitivity to inhibition by d-glucose and d-xylose, make *Pc*AraT a valuable transporter for application in metabolic engineering strategies aimed at engineering *S. cerevisiae* strains for efficient conversion of lignocellulosic hydrolysates.

**Electronic supplementary material:**

The online version of this article (10.1186/s13068-018-1047-6) contains supplementary material, which is available to authorized users.

## Background

At an annual production of 100 Mton [1], bioethanol produced by the yeast *Saccharomyces cerevisiae* is by volume the largest fermentation product in industrial biotechnology. Cane sugar and corn starch, which are still the predominant feedstocks for bioethanol production, almost exclusively yield sucrose and d-glucose as fermentable sugars. Alternative lignocellulosic feedstocks, derived from agricultural residues or energy crops, contain cellulose, hemicellulose, and in some cases, pectin [2]. The pentoses d-xylose and l-arabinose typically represent 10–25 and 2–3%, respectively, of the monomeric sugars in lignocellulosic hydrolysates [3]. Some industrially relevant hydrolysates, however, contain higher l-arabinose concentrations. For instance, in hydrolysates of corn fibre and sugar beet pulp, l-arabinose represents 16 and 26% of the total sugar content, respectively [4, 5].

Whilst pentose sugars are not natural substrates of *S. cerevisiae*, their efficient conversion to ethanol and, ultimately, other bulk products, is essential to ensure economically viable processes [6]. Extensive metabolic and evolutionary engineering has been applied to enable efficient xylose fermentation, based on expression of either a heterologous xylose reductase and xylitol dehydrogenase, or a heterologous xylose isomerase (reviewed by [7] and [8]). Construction of yeast strains capable of l-arabinose fermentation involved functional expression of bacterial genes encoding l-arabinose isomerase (AraA), l-ribulokinase (AraB), and l-ribulose-5-phosphate-4-epimerase (AraD) [9–13]. Additional overexpression of *S. cerevisiae* genes encoding enzymes of the non-oxidative pentose phosphate pathway (*RPE1*, *RKI1*, *TAL1*, and *TKL1*) strongly improved rates of d-xylose and l-arabinose fermentation [12, 14]. In *S. cerevisiae* strains whose metabolic pathways have been intensively optimized for pentose fermentation by metabolic and evolutionary engineering, uptake of l-arabinose and d-xylose is an important rate-controlling step [15–17].

Several *S. cerevisiae* plasma membrane hexose-transporter proteins are able to transport d-xylose and/or l-arabinose but invariably exhibit a high *K*_m_ for these pentoses [18–25]. This low affinity causes sluggish pentose conversion (‘tailing’) towards the end of anaerobic batch cultures. Amongst the set of 18 *S. cerevisiae* hexose transporters (Hxt1-17 and Gal2), only the galactose transporter Gal2 and with, much lower activities, Hxt9 and Hxt10 support l-arabinose import [18, 19]. Gal2 has a high affinity for d-glucose and galactose but its affinity for l-arabinose is low (*K*_m_ = 57–371 mM) [19, 26]. Consequently, engineered strains in which l-arabinose transport depends on Gal2 fail to grow at low l-arabinose concentrations [19]. Moreover, even when d-glucose-induced transcriptional repression of *GAL2* [27–29] is prevented, kinetic competition prevents l-arabinose consumption by such strains in the presence of d-glucose.

So far, few heterologous l-arabinose transporters have been functionally expressed and characterized in *S. cerevisiae* [19, 26, 30]. In these previous studies, *S. cerevisiae* strains harbouring a functional l-arabinose fermentation pathway but no native hexose transporters proved to be excellent platforms for characterization of heterologous l-arabinose transporters. In such experiments, transporters from the yeasts *Scheffersomyces stipitis* (*Ss*AraT), *Pichia guilliermondii* (*Pg*Axt1) and from the plant *Arabidopsis thaliana* (*At*Stp2) were shown to support l-arabinose transport in *S. cerevisiae*. These transporters exhibited *K*_m_ values of 0.13–4.5 mM but low transport capacities, whilst also exhibiting severe d-glucose inhibition [19, 26]. Inhibition by d-xylose was only studied for *Pg*Axt1, where it completely blocked l-arabinose uptake [26]. Conversely, l-arabinose transporters from the fungi *Neurospora crassa* (Lat-1) and *Myceliophthora thermophilum* (*Mt*Lat-1) supported high-capacity, low-affinity (*K*_m_ = 58 and 29 mM, respectively) l-arabinose uptake and were also strongly affected by d-glucose inhibition [30]. The strong inhibition of these transporters by d-glucose and/or d-xylose precludes the simultaneous utilization of d-glucose and l-arabinose in *S. cerevisiae* strains depending on these transporters for l-arabinose uptake.

The filamentous fungus *Penicillium chrysogenum* and its genome have been intensively studied in relation to its role in the production of β-lactam antibiotics [31, 32]. *P. chrysogenum* is able to hydrolyse arabinoxylan to l-arabinose by its Axs5 extracellular arabinofuranohydrolase, followed by uptake and metabolism of l-arabinose as a carbon and energy source [33–35]. This ability implies the presence of one or more membrane transporters capable of importing l-arabinose across the plasma membrane of this fungus.

The goal of this study was to explore the *P. chrysogenum* genome for l-arabinose transporters that can be functionally expressed in *S. cerevisiae* and support d-glucose- and d-xylose insensitive, high-affinity transport of l-arabinose. To this end, transcriptomes of l-arabinose-, ethanol- and d-glucose-limited chemostat cultures of *P. chrysogenum* were compared, and putative l-arabinose transporter genes were tested for their ability to support l-arabinose transport upon expression in an *S. cerevisiae* strain engineered for l-arabinose fermentation in which *GAL2* had been deleted. A *P. chrysogenum* transporter identified in this screen, *Pc*AraT, was subjected to more detailed analysis, including kinetic sugar-uptake studies with radiolabelled substrates, in vivo studies on uptake inhibition, and physiological studies with engineered *S. cerevisiae* strains in l-arabinose-limited chemostat cultures.

## Methods

### Microbial strains, growth media and maintenance

All *S. cerevisiae* strains constructed and used in this study (Table 1) are derived from the CEN.PK lineage [36]. Yeast strains were grown on synthetic medium (SM) [37] or on YP medium (10 g L^−1^ Bacto yeast extract, 20 g L^−1^ Bacto peptone). For shake flask cultures on synthetic medium, ammonium sulfate was replaced with urea as nitrogen source to minimize acidification. The resulting SM-urea contained 38 mmol L^−1^ urea and 38 mmol L^−1^ K_2_SO_4_ instead of (NH_4_)_2_SO_4_. SM and YP media were autoclaved at 121 °C for 20 min, or filter-sterilized using 0.2-µm bottle-top filters (Thermo Scientific, Waltham MA). Subsequently, synthetic media were supplemented with 1 mL L^−1^ of a sterile-filtered vitamin solution [37]. SM, SM-urea and YP media were further supplemented with 20 g L^−1^ d-glucose or l-arabinose, by adding concentrated solutions autoclaved at 110 °C for 20 min, yielding SMD or SMA, SMD-urea or SMA-urea and YPD or YPA, respectively. Yeast cultures were grown in 100 mL medium in 500-mL shake flasks at 30 °C and at 200 rpm in an Innova Incubator (New Brunswick Scientific, Edison NJ). Solid SMD, SMA, YPD and YPA contained 1.5% Bacto agar and when indicated, 200 mg L^−1^ G418 (Invivogen, San Diego, CA). Solid medium with ethanol and glycerol as carbon source (YPEG, SMEG, YPEG-G418) contained 2% ethanol and 3% glycerol. Selection and counter selection of the *amdSYM* marker cassette were performed as described previously [38]. *Escherichia coli* strains were grown in 5 mL Lysogeny Broth (10 g L^−1^ Bacto tryptone, 5 g L^−1^ Bacto yeast extract, 5 g L^−1^ NaCl) supplemented with 100 mg L^−1^ ampicillin in 25-mL shake flasks at 37 °C and 200 rpm in an Innova 4000 shaker (New Brunswick Scientific). Before storage at − 80 °C, yeast and *E. coli* cultures were mixed with glycerol (30% v/v). *P. chrysogenum* DS17690 was kindly provided by DSM Anti-infectives (Delft, The Netherlands) and grown in mineral medium (pH 5.5), containing 3.5 g (NH_4_)_2_SO_4_, 0.8 g KH_2_PO_4_, 0.5 g MgSO_4_·7H_2_O and 10 mL of trace element solution (15 g L^−1^ Na_2_EDTA·2H_2_O, 0.5 g L^−1^ Cu_2_SO_4_·5H_2_O, 2 g L^−1^ ZnSO_4_·7H_2_O, 2 g L^−1^ MnSO_4_·H_2_O, 4 g L^−1^ FeSO_4_·7H_2_O, and 0.5 g L^−1^ CaCl_2_·2H_2_O) per litre of demineralized water. The mineral medium was supplemented with 7.5 g L^−1^ d-glucose. Precultivation for chemostat cultures was carried out on mineral medium with 7.5 g L^−1^ d-glucose, 7.5 g L^−1^ l-arabinose, or 5.8 g L^−1^ ethanol as carbon source.

**Table 1:** *Saccharomyces cerevisiae* strains used in this study

Strain	Relevant genotype	References
CEN.PK 113-7D	*MAT*a *URA3 HIS3 LEU2 TRP1 MAL2*-*8c SUC2*	[36]
CEN.PK 113-5D	*MAT*a *ura3*-*52 HIS3 LEU2 TRP1 MAL2*-*8c SUC2*	[36]
CEN.PK102-12A	*MAT*a *ura3*-*52 his3*-*D1 leu2*-*3*,*112 TRP1 MAL2*-*8c SUC2*	[36]
IMX080	CEN.PK102-12A *glk1::SpHis5*, *hxk1::KlLEU2*	[75]
IMX581	CEN.PK113-5D *can1::cas9*-*natNT2*	[44]
IMX486	IMX080 *gal1::cas9*-*amdSYM*	This study
IMX604	IMX486 *ura3*-*52 gre3::pTDH3*-*RPE1*, *pPGK1*-*TKL1*, *pTEF1*-*TAL1*, *pPGI1*-*NQM1*, *pTPI1*-*RKI1*, *pPYK1*-*TKL2*	This study
IMX658	IMX604 *ura3*-*52 gal80::(pTPI*-*AraA*-tCYC1)*9, *pPYK1*-*AraB*-*tPGI1*, *pPGK1*-*AraD*-*tTDH3*	This study
IMX660	IMX658 *hxk2::KlURA3*	This study
IMX728	IMX658 *hxk2::PcaraT*	This study
IMX844	IMX660 *gal2::KanMX*	This study
IMX869	IMX728 *gal2::KanMX*	This study
IMX918	IMX581 *gre3::pTDH3*-*RPE1*, *pPGK1*-*TKL1*, *pTEF1*-*TAL1*, *pPGI1*-*NQM1*, *pTPI1*-*RKI1*, *pPYK1*-*TKL2*	This study
IMX928	IMX918 *gal80::(pTPI*-*AraA*-tCYC1)*9, *pPYK*-*AraB*-*tPGI1*, *pPGK*-*AraD*-*tTDH3*	This study
IMX929	IMX918 *gal80::(pTPI*-*AraA*-tCYC1)*9, *pPYK*-*AraB*-*tPGI1*, *pPGK*-*AraD*-*tTDH3*, pUDE348	This study
IMX1504	IMX928, *gal2Δ*, pUDR245	This study
IMX1505	IMX928 *gal2::pADH1-Pc13g04640-tPMA1* (from pPWT111), pUDR245	This study
IMX1506	IMX928 *gal2::pADH1-Pc13g08230-tPMA1* (from pPWT113), pUDR245	This study
IMX1507	IMX928 *gal2::pADH1-Pc16g05670-tPMA1* (from pPWT116), pUDR245	This study
IMX1508	IMX928 *gal2::pADH1-Pc20g01790-tPMA1 (PcaraT)* (from pPWT118), pUDR246	This study
IMX1509	IMX928 *gal2::pADH1-Pc22g14520-tPMA1* (from pPWT123), pUDR245	This study
DS68616	*MAT*a, *ura3*-*52*, *leu2*-*112*, *gre3::loxP*, *loxP*-*pTPI*-*TAL1*, *loxP*-*pTPI*-*RKI1*, *loxP*-*pTPI*-*TKL1*, *loxP*-*pTPI*-*RPE1*, *leu2::pADH1*-*XKS1*-*tCYC1*-*LEU2*, *ura3::URA3*-*pTPI1*-*XylA*-*tCYC1*	DSM, The Netherlands
DS68625	DS68616 *his3::loxP*, *hxt2::loxP*-*kanMX*-*loxP*, *hxt367::loxP*-*hphMX*-*loxP*, *hxt145::loxP*-*natMX*-*loxP*, *gal2::loxP*-*zeoMX*-*loxP*	[45]
DS68625-*PcaraT*	DS68625, pRS313-*PcaraT*	This study
DS68625-*GAL2*	DS68625, pRS313-*GAL2*	This study
DS68625-mcs	DS68625, pRS313-mcs (empty)	This study

### Molecular biology techniques

DNA fragments were amplified by PCR amplification with Phusion Hot Start II High Fidelity Polymerase (Thermo Scientific) and desalted or PAGE-purified oligonucleotide primers (Sigma-Aldrich, St. Louis, MO) performed according to the manufacturers’ instructions. Diagnostic PCRs were run with DreamTaq polymerase (Thermo Scientific). Oligonucleotide primers used in this study are listed in Additional file 1. PCR products were separated by electrophoresis on 1% (w/v) agarose gels (Thermo Scientific) in TAE buffer (Thermo Scientific) and, if required, purified with a Zymoclean Gel DNA Recovery kit (Zymo Research, Irvine, CA) or a GenElute PCR Clean-Up kit (Sigma-Aldrich). Yeast or *E. coli* plasmids were isolated with a Zymoprep Yeast Plasmid Miniprep II kit (Zymo Research), or a Sigma GenElute Plasmid kit (Sigma-Aldrich), respectively. A YeaStar Genomic DNA kit (Zymo Research) or an SDS/lithium acetate protocol [39] was used to isolate yeast genomic DNA. Yeast strains were transformed using the lithium acetate/polyethylene glycol method [40]. Single-colony isolates were obtained from three consecutive re-streaks on selective solid agar plates, followed by analytical PCR analysis of the relevant genotype. *E. coli* DH5α cultures were transformed by chemical transformation [41]. After isolation, plasmids were verified by restriction analysis and analytical PCR.

### Plasmid construction

Plasmids used in this study are shown in Table 2. All synthesized gene expression cassettes were constructed by GeneArt (Regensburg, Germany). Genes encoding the five putative transporters Pc13g04640 [Genbank: CAP91533.1], Pc13g08230 [Genbank: CAP91892.1], Pc16g05670 [Genbank: CAP93237.1], Pc20g01790 (*PcaraT*) [Genbank: CAP85508.1] and Pc22g14520 [Genbank: CAP98740.1] were codon-pair optimized [42] for expression in *S. cerevisiae* and cloned into the plasmid pPWT007 [43] resulting in pPWT111, 113, 116, 118 and 123, respectively, harbouring each an expression cassette consisting of the *ADH1* promoter, the codon-optimized open-reading frame of a putative transporter gene, and the *PMA1* terminator. Expression cassettes for the coding regions of *Lactobacillus plantarum* l-arabinose isomerase *araA* [Genbank: ODO63149.1], l-ribulose kinase *araB* [Genbank: ODO63147.1] and l-ribulose-5P epimerase *araD* [Genbank: ODO63148.1] were codon-optimized using the most common codons present in the glycolytic genes of *S. cerevisiae* [10] and provided by GeneArt in pMK-RQ-based cloning vectors named, pUDE354, pUDE355 and pUDE356, respectively. The episomal plasmids used to express guide RNAs (gRNAs) were constructed from PCR amplified fragments that were ligated using the Gibson Assembly Cloning kit (New England Biolabs, Ipswich, MA). gRNA plasmids pUDR246 and pUDR245 were constructed using pROS10 as a template [44], with oligonucleotide primers listed in Additional file 1. pUDE348 was derived from pMEL10 by first PCR amplifying the plasmid backbone using primers 5792 and 5980. The gRNA sequence was introduced in the gRNA expression cassette with primers 6631 and 5979 using pMEL10 [44] as a template. Subsequently, both fragments were combined using the Gibson Assembly Cloning kit. pUD405 was obtained by integration of a Gal2-flanked KanMX cassette obtained from pUG6 with primers 944 and 945 into a pJET1.2 blunt vector according to the manufacturers’ instructions. Construction of the low-copy-number centromeric plasmid pRS313-mcs was described previously [45]. *GAL2* was amplified from genomic DNA of *S. cerevisiae* DS68616 [45] and *PcaraT* was amplified from plasmid pPWT118 using primers F *GAL2* Xbai and R *GAL2* Cfr9i and primers F *PcaraT* Xbai and R *PcaraT* Cfr9i, respectively, and cloned into pRS313-mcs, resulting in plasmids pRS313-*PcaraT* and pRS313-*GAL2*.

**Table 2:** Plasmids used in this study

Plasmid	Characteristics	Source
p414-*TEF1p*-*Cas9*-*CYC1t*	*CEN6/ARS4* ampR *pTEF1*-*cas9*-*tCYC1*	[76]
pUG-*amdSYM*	Template for *amdSYM* marker	[38]
pUG-72	Template for *KlURA3* marker	[77]
pUG6	Template for *KanMX* marker	[78]
pUDE327	2 μm, *KlURA3*, *pSNR52*-gRNA.*HXK2.Y*	[79]
pUDE335	2 μm, *KlURA3*, *pSNR52*-gRNA*.GRE3*.Y	[50]
pUDE348	2 μm, *KlURA3*, *pSNR52*-gRNA.*GAL80.Y*	This study
pUDR246	2 μm, *KlURA3*, *pSNR52*-gRNA*.GAL2.Y pSNR52*-gRNA*.GAL2.Y*	This study
pUDR245	2 μm, *KlURA3*, *pSNR52*-gRNA*.GAL2.Y pSNR52*-gRNA*.GAL2.Y*	This study
pMEL10	*pSNR52*-gRNA.*CAN1.Y*-*tSUP4*	[44]
pROS10	2 μm, *KlURA3*, *pSNR52*-gRNA.*CAN1*.*Y pSNR52*-gRNA.*ADE2.Y*	[44]
pUD344	pJET1.2Blunt TagA-*pPGI1*-*NQM1*-TagB	[50]
pUD345	pJET1.2Blunt TagB-*pTPI1*-*RKI1*-TagC	[50]
pUD346	pJET1.2Blunt TagC-*pPYK1*-*TKL2*-TagF	[50]
pUD347	pJET1.2BluntTagG-*pTDH3*-*RPE1*-TagH	[50]
pUD348	pJET1.2Blunt TagH-*pPGK1*-*TKL1*-TagI	[50]
pUD349	pJET1.2Blunt TagI-*pTEF1*-*TAL1*-TagA	[50]
pUD405	pJET1.2Blunt *GAL2 flanked KanMX*	This study
pPWT111	ampR *KanMX*, *amdSYM*, *pADH1*-Pc13g04640-*tPMA1*	This study
pPWT113	ampR *KanMX*, *amdSYM*, *pADH1*-Pc13g08230-*tPMA1*	This study
pPWT116	ampR *KanMX*, *amdSYM*, *pADH1*-Pc16g05670-*tPMA1*	This study
pPWT118	ampR *KanMX*, *amdSYM*, *pADH1*-Pc20g01790 *(PcaraT)*-*tPMA1*	This study
pPWT123	ampR *KanMX*,* amdSYM*, *pADH1*-Pc22g14520-*tPMA1*	This study
pUD354	*pMK*-*RQ*-*pTPI1*-*araA*-*tADH3*	This study
pUD355	*pMK*-*RQ*-*pPYK1*-*araB*-*tPGI1*	This study
pUD356	*pMK*-*RQ*-*pPGK1*-*araD*-*tTDH3*	This study
pRS313-mcs	*CEN6*, *ARSH4*, *HIS3*-*pHXT7*, *tHXT7*	[45]
pRS313-*PcaraT*	*CEN6*, *ARSH4*, *HIS3*, ampR, *pHXT7*-*PcaraT*-*tHXT7*	This study
pRS313-*GAL2*	*CEN6*, *ARSH4*, *HIS3*, ampR, *pHXT7*-*GAL2*-*tHXT7*	This study

### Strain construction

Gene expression cassettes were PCR amplified with oligonucleotide primers shown in Additional file 1 and genomic DNA of CEN.PK113-7D or plasmids described in Table 2. Gene knock-outs and construct integrations were introduced with a chimeric CRISPR/Cas9 editing system [44]. To enable CRISPR/Cas9 mediated editing in strain IMX080, the *Sp*Cas9 expression cassette was amplified from p414-*pTEF1*-*cas9*-*tCYC1* (Addgene plasmid # 43802) and integrated into the *GAL1* locus via in vivo assembly, together with the *amdSYM* marker, yielding strain IMX486. For overexpression of the non-oxidative pentose phosphate pathway (PPP), IMX486 and IMX581 were co-transformed with gRNA plasmid pUDE335 and repair fragments flanked with either 60 bp homologous to *GRE3* or with synthetic tags [46] assisting homologous recombination of the PPP expression cassettes (*gre3*_*flank*_-*pTDH3*-*RPE1*-*TagH*, *TagH*-*pPGK1*-*TKL1*-*TagI*, *TagI*-*pTEF1*-*TAL1*-*TagA*, *TagA*-*pPGI1*-*NQM1*-*TagB*, *TagB*-*pTPI1*-*RKI1*-*TagC*, *TagC*-*pPYK1*-*TKL2*-*gre3*_*flank*_). After counter selection of the *URA3*-based plasmid pUDE335, the resulting strains, IMX604 and IMX918, respectively, were co-transformed with pUDE348 and repair fragments flanked with either 60 bp homologous to *GAL80* or with synthetic tags [46] (*GAL80*_*flank*_-*pTPI1*-*araA*-*TagG*, *TagG*-*pTPI1*-*araA*-*TagA*, *TagA*-*pTPI1*-*araA*-*TagB*, *TagB*-*pTPI1*-*araA*-*TagC*, *TagC*-*pTPI1*-*araA*-*TagD*, *TagD*-*pTPI1*-*araA*-*TagM*, *TagM*-*pTPI1*-*araA*-*TagN*, *TagN*-*pTPI1*-*araA*-*TagO*, *TagO*-*pTPI1*-*araA*-*TagI*, *TagI*-*pPYK1*-*araB*-*TagK*, *TagK*-*pPGK1*-*araD*-*GAL80*_*flank*_) resulting in nine copies of *araA* and a single copy of *araB* and *araD* integrated in the *GAL80* locus. After verification of the resulting strains IMX929 and IMX658, respectively, plasmid pUDE348 was counter selected in IMX929 to yield strain IMX928. Disruption of *HXK2* in IMX658 was done by PCR amplification and transformation of the *KlURA3*-based deletion cassette from pUG-72 [76] to obtain strain IMX660 upon transformation and plating in solid SMA. *GAL2* was disrupted in IMX660 by transformation with a KanMX cassette amplified from pUD405 with primers 944 and 945 flanked with 60 bp homologous to *GAL2*. Transformants were incubated for 2 h in YPE before plating on YPEG-G418, yielding strain IMX844. Expression of *PcaraT* in IMX658 was achieved by transforming IMX658 with the gRNA plasmid pUDE327 together with an expression cassette of *PcaraT* (pADH1-*PcaraT*-tPMA1) with flanking regions homologous to the *HXK2* locus amplified with the primer pair 7660 and 7676. Counter selection of the pUDE327 and subsequent transformation of a DNA fragment derived from CEN.PK113-7D using primers 2641 and 1522 repaired uracil auxotrophy and resulted in strain IMX728. *GAL2* was disrupted in IMX728 by transformation with a KanMX cassette amplified from pUD405 with primers 944 and 945 flanked with 60 bp homologous to *GAL2*. Transformants were incubated for 2 h in YPE before plating on YPEG-G418, yielding strain IMX869. Strains IMX1505-1509 were constructed by co-transforming pUDR245 or pUDR246 and a *GAL2*-flanked expression cassette (pADH1-ORF-tPMA1) amplified from pPWT111, 113, 116, 118 or 123, respectively, amplified with the primer pair 10585 and 10584. IMX1504, harbouring a knockout of *GAL2*, was constructed by co-transforming pUDR245 and a repair fragment based on the annealed primers 9563 and 9564. Transformation of *GAL2* and *PcaraT* plasmids, and the pRS313-mcs plasmid (as an empty plasmid/control) into the hexose-transporter deletion strain DS68625 yielded strains DS68625-*GAL2*, DS68625-*PcaraT*, and DS68625-mcs.

### Growth experiments in shake flasks

Thawed 1-mL aliquots from frozen stock cultures were used to inoculate shake flask precultures on SM-urea supplemented with either d-glucose (20 g L^−1^), l-arabinose (20 g L^−1^), or both sugars (both 20 g L^−1^). These precultures were used to inoculate a second culture which was subsequently used to inoculate a third culture which was inoculated at an initial OD_660_ of 0.1 and used to monitor growth. Optical densities at 660 nm were measured with a Libra S11 spectrophotometer (Biochrom, Cambridge, United Kingdom). Maximum specific growth rates (*μ*_max_) were derived from at least four consecutive data points derived from samples taken during the exponential growth phase of each culture.

### Spot plates

l-Arabinose-metabolizing *S. cerevisiae* strains expressing putative *P. chrysogenum* l-arabinose transporter genes (IMX1504-1509) were grown on SMD medium and a total number of approximately 10^4^, 10^3^, 10^2^, and 10^1^ cells were spotted on duplicate agar plates as described previously [47, 48] containing either 20 g L^−1^ l-arabinose or d-glucose as carbon source (pH 6). Cell numbers were estimated from calibration curves of OD_660_ versus cell counts determined with an Accuri flow cytometer (Becton–Dickinson B.V., Breda, The Netherlands), derived from exponentially growing shake flask cultures of *S. cerevisiae* CEN.PK113-7D on SMD medium. SMA and SMD plates were incubated at 30 °C for 97 and 41 h, respectively.

### Chemostat cultivation

Aerobic carbon-limited chemostat cultures of *P. chrysogenum* were grown at 25 °C in 3-L turbine-stirred bioreactors (Applikon, Schiedam, The Netherlands) with a working volume of 1.8 L and a dilution rate of 0.03 h^−1^ as described previously [49], with the exception that, in addition to cultures grown on 7.5 g L^−1^ d-glucose, chemostat cultures were also grown on either 7.5 g L^−1^ l-arabinose or 5.8 g L^−1^ ethanol. Aerobic, l-arabinose-limited chemostat cultures of *S. cerevisiae* were grown at 30 °C in 2-L Applikon bioreactors with a working volume of 1 L and at a dilution rate of 0.05 h^−1^. SMA (7.5 g L^−1^ l-arabinose) supplemented with 0.15 g L^−1^ Pluronic antifoam PE 6100 was used as culture medium for the initial batch phase and for chemostat cultivation, with the exception of the initial batch phase of strain IMX929 which was grown on 20 g L^−1^ l-arabinose. Cultures were stirred at 800 rpm, kept at pH 5.0 by automatic addition of 2 M KOH, and sparged with 0.5 L min^−1^ air. Upon completion of the batch phase, chemostat cultivation was initiated, ensuring a constant culture volume with an electric level sensor. When after at least five volume changes, biomass dry weight and CO_2_ production varied by less than 2% over two consecutive volume changes, the culture was considered to be in steady state.

### Analytical methods

*Penicillium chrysogenum* biomass dry weight was determined in duplicate by filtration of 10 mL culture sample over pre-weighed glass fibre filters (Type A/E, Pall Life Sciences, Hoegaarden, Belgium). After filtration, filters were washed with demineralized water and dried for 10 min at 600 W in a microwave oven (Bosch, Stuttgart, Germany) prior to reweighing. Biomass dry weight in *S. cerevisiae* culture samples was determined with a similar procedure using nitrocellulose filters (0.45-µm pore size; Gelman Laboratory, Ann Arbor, MI) and drying for 20 min in a microwave oven at 360 W output. Optical density (OD) of the cultures was determined at 660 nm with a Libra S11 spectrophotometer (Biochrom, Cambridge, United Kingdom). Determination of CO_2_ and O_2_ concentrations in the bioreactor exhaust gas and HPLC analysis of metabolite concentrations in culture supernatant samples were performed as described previously [50].

### Sampling, RNA extraction, microarrays analysis, and data analysis

Samples (60 mL) from *P. chrysogenum* chemostat cultures were rapidly filtered over a glass fibre filter (Type A/E, Pall Life Sciences) and further processed for total RNA extraction by phenol–chloroform extraction [49]. The cRNA sample preparation (cDNA synthesis, purification, in vitro transcription, labelling, purification, fragmentation and biotinylation) was performed according to Affymetrix recommendations [31]. Eventually cRNA samples were hybridized onto custom-made *P. chrysogenum* GeneChip microarrays (array code DSM_PENa520255F). Data acquisition, hybridization, quantification of processed array images, and data filtering were performed using the Affymetrix GeneChip Operating Software (GCOS version 1.2). Global array normalization was performed by scaling the global fluorescence intensity of each microarray to 100. The scaling factors of the individual arrays were highly similar and ranged from 0.21 to 0.35. Subsequently, significant variations in expression were statistically estimated by comparing replicate array experiments using the Significance Analysis of Microarray software (SAM version 2.0) [51] with the multiclass setting. A false discovery rate of 1% was applied to minimize the chance of false-positive hits. Genes with an over threefold higher transcript level in arabinose-grown cultures than in d-glucose-grown cultures and a less than threefold difference in ethanol- and d-glucose-grown cultures were deemed to show arabinose-specific expression. Transcriptome data of strain DS17690 grown on d-glucose, ethanol or arabinose are accessible at NCBI Genome Omnibus database (https://www.ncbi.nlm.nih.gov/geo/) under Accession Numbers GSE12632, GSE24212 and GSE10449, respectively [49].

### Analysis of sugar uptake kinetics

Uptake experiments with [^14^C] l-arabinose, [^14^C] d-xylose, or [^14^C] d-glucose, labelled at the first carbon atom (50–60 mCi/mmol) (ARC St. Louis, MO), were performed with *S. cerevisiae* hexose-transporter deletion strains (DS68625) harbouring a low copy plasmid with constitutively expressed *PcaraT* (pRS313-*PcaraT*) or *GAL2* (pRS313-*GAL2*). The experimental workflow was carried out as described previously [45] with [^14^C] l-arabinose concentrations of 0.5–2000 mmol L^−1^, [^14^C] d-xylose concentrations of 0.5–500 mM, or [^14^C] d-glucose concentrations of 0.1–500 mmol L^−1^. Transport competition experiments were carried out in the presence of 50 mmol L^−1^ [^14^C] l-arabinose and 0–500 mmol L^−1^ d-glucose or d-xylose, and at [^14^C] l-arabinose concentration of 2 mmol L^−1^ together with increasing d-glucose and xylose concentrations of 0–20 mM. Maximum biomass-specific transport rates (‘*V*_max_’) calculated from transport assays were expressed as nmol sugar transported per milligram biomass dry weight per minute [nmol (mg biomass)^−1^ min^−1^]. As this *V*_max_ is influenced by the expression level of the relevant transporter, it is not solely dependent on intrinsic transporter kinetics. The impact of proton-gradient uncoupling on transport activity was determined in 200 μL synthetic medium at a [^14^C]-l-arabinose concentration of 2 mmol L^−1^, by comparing transport rates upon addition of either 10 μmol L^−1^ CCCP (0.5 µL of a stock solution dissolved in 100% DMSO), 0.5 μL DMSO (control), or 0.5 µL water.

### Phylogenetic methods

Protein sequences used for generation of a phylogenetic tree were derived from NCBI (https://www.ncbi.nlm.nih.gov/) and the *Saccharomyces* Genome Database (https://www.yeastgenome.org/). Mafft was used to generate a CLUSTAL format alignment of all sequences, using the L-INS-i method default settings (https://mafft.cbrc.jp/alignment/server/) [52, 53]. Alignments were further processed using neighbour-joining and a 500 times bootstrap. The resulting Newick tree file was visualized and midpoint rooted in iTOL (https://itol.embl.de/) [54]. Gene accession numbers were *ScGAL2*: P13181, *PcaraT*: CAP85508, *SsaraT*: XP_001382755, *Atstp2*: OAP13698, *Kmaxt1*: GZ791039, *Pgaxt1*: GZ791040, *Amlat1*: AY923868, *Amlat2*: AY923869, *Nclat*-*1*: EAA30346, *Mtlat*-*1*: XP_003663698.

## Results

### Chemostat-based transcriptome analysis of *P. chrysogenum* for identification of possible l-arabinose transporter genes

Filamentous fungi exhibit a much broader range of carbon source utilization than *S. cerevisiae* and, similar to many other ascomycetous fungi, *P. chrysogenum* can grow on l-arabinose as the sole carbon source [33, 55]. To identify candidate structural genes for l-arabinose transporters in *P. chrysogenum*, carbon-limited chemostat cultures of strain DS17690 were grown at a dilution rate of 0.03 h^−1^ on different carbon sources. To discriminate between alleviation of carbon repression and l-arabinose induction, duplicate d-glucose-, l-arabinose-, and ethanol-limited chemostat cultures were performed. RNA was extracted from steady-state cultures and gene expression levels were obtained using Affymetrix DNA-arrays [49]. A total of 540 genes were differentially expressed over the three conditions. Of these differentially expressed genes, 137 exhibited an over threefold higher transcript level in l-arabinose-limited cultures than in d-glucose-limited cultures, as well as a less than threefold difference in transcript level between ethanol- and d-glucose-limited cultures (Additional file 2). Genes whose transcript levels in l-arabinose- and ethanol-limited cultures were both at least twofold higher than in d-glucose-grown cultures were not considered for further analysis as their regulation could have reflected unspecific d-glucose (de)repression. An annotation screen indicated that 16 of the identified ‘arabinose-induced’ genes encoded putative transporters, whose transcript levels were 3.4- to 52-fold higher in the l-arabinose-limited cultures than in the d-glucose-limited cultures (Table 3). Five of these genes, whose transcript levels were at least 30-fold higher in l-arabinose-limited cultures than in d-glucose-limited cultures, shared similarity with the *S. cerevisiae* maltose transporter Mal31, the *N. crassa* d-glucose transporter Rco-3, the *Kluyveromyces lactis* high-affinity d-glucose transporter Hgt1 and the *S. cerevisiae* allantoate transporter Dal5. These five transporter genes (Pc13g08230, Pc16g05670, Pc20g01790, Pc22g14520, and Pc13g04640, respectively) were selected for further functional analysis.

**Table 3:** Putative transporter genes that showed higher relative transcript levels in aerobic, l-arabinose-limited chemostat cultures of *Penicillium chrysogenum* than in corresponding d-glucose- and ethanol-limited cultures

Gene	Strong similarity to	Relative transcript levels under different nutrient limitations				
		Glucose	l-Arabinose	Ethanol	Ethanol versus glucose (ratio)	l-Arabinose versus glucose (ratio)
Pc13g08230	*S. cerevisiae* maltose transport protein Mal31	13 ± 1	664 ± 3	17 ± 1	1.4	53
Pc16g05670	*Neurospora crassa* glucose transporter rco-3	63 ± 28	3176 ± 40	69 ± 1	1.1	51
Pc20g01790 ( *PcaraT* )	*Kluyveromyces lactis* high-affinity glucose transporter HGT1	32 ± 6	1415 ± 42	46 ± 3	1.4	44
Pc22g14520	*S. cerevisiae* allantoate permease Dal5	19 ± 2	770 ± 104	28 ± 1	1.5	41
Pc13g04640	*K. lactis* high-affinity glucose transporter HGT1	29 ± 5	971 ± 32	53 ± 7	1.8	34
Pc21g10190	*K. lactis* high-affinity glucose transporter HGT1	12 ± 1	167 ± 26	12 ± 1	1.0	14
Pc12g00190	*Candida albicans* ABC transporter CDR4	13 ± 2	164 ± 24	29 ± 2	2.2	12
Pc14g01680	*Escherichia coli* l-fucose permease fucP	106 ± 14	1269 ± 172	68 ± 1	0.64	12.0
Pc21g12210	*Aspergillus nidulans* quinate transport protein qutD	12 ± 0	118 ± 1	12 ± 1	1	9.8
Pc06g01480	*S. cerevisiae* maltose transport protein Mal31	459 ± 85	3551 ± 102	226 ± 3	0.5	7.7
Pc13g10030	*S. cerevisiae* high-affinity nicotinic acid permease Tna1	125 ± 25	827 ± 33	216 ± 3	1.7	6.6
Pc21g09830	*K. lactis* high-affinity glucose transporter HGT1	185 ± 9	842 ± 1	126 ± 3	0.68	4.6
Pc16g02680	*S. cerevisiae* allantoate permease Dal5	80 ± 29	360 ± 14	113 ± 6	1.4	4.5
Pc12g05440	*S. cerevisiae* maltose transport protein Mal31	596 ± 201	2633 ± 64	104 ± 8	0.17	4.4
Pc13g15590	*S. cerevisiae* glucose permease Rgt2	12 ± 1	48.0 ± 1.0	12 ± 1	1	4.0
Pc13g06440	*S. cerevisiae* high-affinity nicotinic acid permease Tna1	66 ± 23	225 ± 11	48 ± 5	0.73	3.4

*P. chrysogenum* DS1769 was grown in l-arabinose-, d-glucose-, or ethanol-limited chemostat cultures (dilution rate = 0.03 h^−1^, pH 6.5, *T* = 25 °C). Underlined genes were selected for further analysis based on a ≥ 30-fold higher transcript level in l-arabinose-limited cultures than in d-glucose-limited cultures. Data represent average ± mean deviation of globally scaled (target 100) Affymetrix microarrays for independent duplicate chemostat cultures

### *Pc*AraT: a *P. chrysogenum*l-arabinose transporter that can be functionally expressed in *S. cerevisiae*

**Fig. 1:**
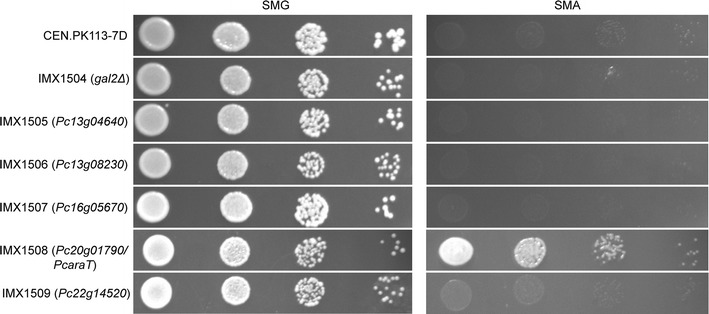
Impact of the expression of putative *P. chrysogenum* sugar transporter genes in an l-arabinose metabolizing *S. cerevisiae* strain in which *GAL2* was deleted. Strains were pregrown on liquid SMD and spotted on plates containing 20 g L^−1^ d-glucose (SMD, left) or l**-**arabinose (SMA, right) as carbon source. Codes on left-hand side indicate *S. cerevisiae* strain names and, in brackets, the systematic name of the corresponding over-expressed *P. chrysogenum* gene. CEN.PK113-7D is a control strain that was not engineered for l-arabinose metabolism. SMD and SMA plates were incubated at 30 °C for 47 and 91 h, respectively. The experiment was performed in duplicate; data shown are from a single representative experiment

*Saccharomyces cerevisiae* strains in which *HXT* transporter genes have been deleted and which express heterologous pathways for pentose metabolism have proven to be powerful platforms for screening and characterization of heterologous pentose transporter genes [19, 26, 56]. To enable screening for *P. chrysogenum* l-arabinose transporters, *S. cerevisiae* strains were first engineered for l-arabinose consumption. Using CRISPR/Cas9-mediated in vivo assembly [44], the overexpression cassettes for all structural genes involved in the non-oxidative pentose phosphate pathway (*TAL1*, *NQM1*, *TKL1*, *TKL2*, *RKI1*, *RPE1*) were stably integrated into the *GRE3* locus, thereby inactivating synthesis of the Gre3 aldose reductase. Subsequently, nine copies of an expression cassette for overexpression of codon-optimized *L. plantarum* l-arabinose isomerase AraA and single copies of *L. plantarum* AraB (l-ribulokinase) and AraD (l-ribulose-5-phosphate-4-epimerase) expression cassettes were integrated into the *GAL80* locus, using a strain construction strategy previously described for expression of a d-xylose pathway into *S. cerevisiae* [50]. This integration inactivated *GAL80* and thereby alleviated transcriptional repression by d-glucose of *GAL2*, which encodes the major l-arabinose transporter in *S. cerevisiae* [57, 58]. The resulting strain IMX929 was able to grow in liquid media supplemented with l-arabinose as the sole carbon source and was used as a platform strain to test if any of the five selected putative *P. chrysogenum* transporter genes, placed under the control of the constitutive *ADH1* promoter, could support l-arabinose transport in *S. cerevisiae*. To this end, single copies of codon-optimized expression cassettes were integrated into the *GAL2* locus of the l-arabinose-metabolizing *S. cerevisiae* strain IMX928, a uracil auxotrophic daughter strain of IMX929, thereby inactivating the *GAL2* gene. Consistent with previous studies [19, 26], inactivation of *GAL2* in the l-arabinose metabolizing strain IMX928 yielded a strain (IMX1504) that was unable to grow on SMA plates (Fig. 1). All five strains in which *GAL2* had been replaced by putative *P. chrysogenum* transporter genes (IMX1505-1509) showed vigorous growth on SMD plates. However, only strain IMX1508, which expressed the *P. chrysogenum* gene Pc20g01790, showed growth on l-arabinose (Fig. 1). Based on this observation, Pc20g01790 was designated *PcaraT* (*P. chrysogenum* Arabinose Transporter). A Blast-p search revealed strong homology of Pc20g01790 with the *K. lactis* gene *HGT1*, which encodes a high-affinity d-glucose and galactose transporter [59, 60].

### *PcaraT* encodes a high-affinity, high-specificity l-arabinose transporter

Sugar transport kinetics of *Pc*AraT were analysed using ^14^C-labelled l-arabinose, d-xylose and d-glucose. To dissect transporter kinetics of *Pc*AraT and Gal2, their structural genes were separately expressed in *S. cerevisiae* DS68625 [45]. Each gene was introduced on a centromeric plasmid and expressed from the *HXT7* promoter. In strain DS68625, the major hexose-transporter genes (*HXT1*-*7* and *GAL2*) are deleted, whilst its inability to metabolize l-arabinose enables the specific analysis of sugar uptake rather than the combination of radioactive sugar uptake and metabolism. The negative control strain DS68625-mcs (DS68625 transformed with the ‘empty’ centromeric plasmid pRS313-mcs) did not show significant [^14^C] l-arabinose uptake, whilst expression of either Gal2 or *Pc*AraT (strains DS68625-*GAL2* and DS68625-*PcaraT*, respectively) restored l-arabinose transport (Table 4). In kinetic analyses, the *K*_m_ of *Pc*AraT for l-arabinose (0.13 mmol L^−1^) was found to be three orders of magnitude lower than that of Gal2 (335 mmol L^−1^), whilst its transport capacity (*V*_max_) was 14-fold lower than that of Gal2 [5.3 and 75 nmol (mg biomass)^−1^ min^−1^, respectively] (Table 4). *Pc*AraT was found to be highly l-arabinose specific, as its expression in strain DS68625 did not support transport of either [^14^C] d-glucose or [^14^C] d-xylose. Consistent with earlier reports [19, 26], expression of Gal2 in strain DS68625 enabled transport of d-glucose [*K*_m_ = 1.9 mmol L^−1^, *V*_max_ = 26 nmol (mg biomass)^−1^ min^−1^], whilst Gal2 has previously been shown to enable low-affinity d-xylose transport (*K*_m_ = 226 mmol L^−1^; [20]).

**Table 4:** Kinetic data for the *S. cerevisiae* transporter Gal2 and *P. chrysogenum Pc*AraT derived from uptake studies with ^14^C-labelled l-arabinose, d-glucose and d-xylose. Sugar transport kinetics were measured by uptake of ^14^C**-**radiolabelled sugars by *S. cerevisiae* DS68625, an engineered strain lacking the Hxt1-7 and Gal2 transporters, expressing either *GAL2* or *PcaraT*

	Gal2	*Pc*AraT
*K*_m, ARA_ (mmol L^−1^)	335 ± 21	0.13 ± 0.03
*V*_max, ARA_ [nmol (mg biomass)^−1^ min^−1^]	75 ± 5.2	5.3 ± 0.2
*K*_m, GLC_ (mmol L^−1^)	1.9	–
*V*_max, GLC_ [nmol (mg biomass)^−1^ min^−1^]	26	–
l-Arabinose transport inhibition by glucose	85%	63%
*K*_m, XYL_ (mmol L^−1^)	226 [20]	–
*V*_max, XYL_ [nmol (mg biomass)^−1^ min^−1^]	91 [20]	–
l-Arabinose transport inhibition by d-xylose	29%	22%

Transport inhibition was determined at 50 mmol L^−1^ [^14^C] l-arabinose and 100 mmol L^−1^ of either d-glucose or d-xylose and expressed relative to the transport rate observed in the absence of d-xylose or d-glucose. Values are represented as average ± mean deviation of duplicate experiments. Graphs used to calculate kinetic parameters are shown in Additional files 3–6. ARA, l-arabinose; GLC, d-glucose; XYL, d-xylose; –, no transport

The impact of the presence of d-glucose and d-xylose on l-arabinose transport by Gal2 and *Pc*AraT was investigated in transport assays with 50 mmol L^−1^ [^14^C] l-arabinose and increasing concentrations of non-radioactive d-glucose or d-xylose. In these assays, both transporters exhibited a reduced l-arabinose transport capacity in the presence of d-glucose or d-xylose (Table 4, Additional file 3). At a concentration of 100 mmol L^−1^ (i.e. twice the concentration of l-arabinose), d-xylose and d-glucose inhibited l-arabinose uptake rate via Gal2 by 29 and 85%, respectively. In contrast, l-arabinose transport via *Pc*AraT was less impaired at this concentration of d-xylose, and especially, d-glucose (22 and 63% inhibition, respectively). To study the transport mechanism of *Pc*AraT, the impact of the protonophore uncoupler CCCP on transport kinetics was tested. Transport of l-arabinose via Gal2, which mediates facilitated diffusion of sugars [61], was not affected by CCCP, whilst this uncoupler completely abolished transport via *Pc*AraT (Additional file 7). These results indicate that *Pc*AraT mediates proton-coupled import of l-arabinose.

### Functional expression of *PcaraT* in an l-arabinose-fermenting *S. cerevisiae* strain enables l-arabinose consumption in the presence of d-glucose

**Fig. 2:**
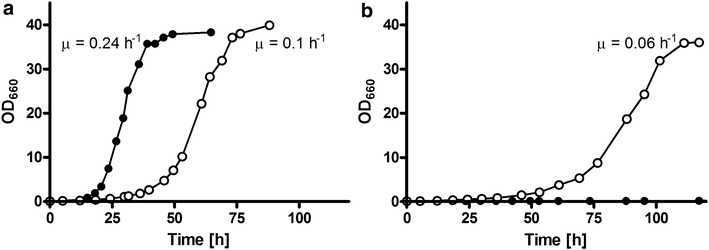
Growth curves of *S. cerevisiae* l-arabinose specialist strains, engineered for l-arabinose consumption and disabled for d-glucose consumption by deletion of the hexose kinase genes *HXK1*, *HXK2*, *GLK1* and *GAL1*, and expressing either *GAL2* (IMX660, filled circles) or the *P. chrysogenum* transporter *Pc*AraT (IMX869, open circles) as the sole l-arabinose transporter. To assess the ability of Gal2 and *Pc*AraT to support import of l-arabinose by growing cultures in the absence (**a**) and presence (**b**) of d-glucose, specific growth rates were estimated from shake flask cultures on synthetic media supplied with 20 g L^−1^ l-arabinose (**a**) and on synthetic media supplied with l-arabinose and d-glucose (20 g L^−1^ each, **b**)

The ability to transport l-arabinose in the presence of d-glucose is a highly relevant characteristic in the construction of platform *S. cerevisiae* strains for conversion of lignocellulosic hydrolysates [8]. To investigate whether expression of *PcaraT* can confer this ability, a set of three strains was constructed that (i) could not metabolize d-glucose due to the deletion of *HXK1*, *HXK2*, *GLK1* and *GAL1* [20, 62]; (ii) (over)expressed non-oxidative PPP enzymes and the *L. plantarum* AraA, AraB and AraD genes to enable l-arabinose metabolism; and (iii) had different genotypes with respect to l-arabinose transport (*GAL2*, *PcaraT*/*gal2Δ* and *gal2Δ* in strains IMX660, IMX869 and IMX844, respectively). Since these ‘arabinose specialist strains’ cannot grow on d-glucose, the impact of the presence of d-glucose on l-arabinose metabolism can be directly measured via its effect on growth. As anticipated, strain IMX844 (*gal2*Δ) was unable to grow on synthetic medium supplemented with either 20 g L^−1^ l-arabinose or a mix of 20 g L^−1^ of each, l-arabinose and d-glucose. In contrast, the l-arabinose specialist strains IMX660 (*GAL2*) and IMX869 (*PcaraT*/*gal2Δ*) grew on synthetic medium with l-arabinose as the sole carbon source at specific growth rates of 0.240 ± 0.001 and 0.099 ± 0.001 h^−1^, respectively (Fig. 2a). However, when 20 g L^−1^ d-glucose was added to the l-arabinose medium, strain IMX660 (*GAL2*) did not show growth during a 120-h batch cultivation experiment (Fig. 2b), whilst strain IMX869 (*PcaraT*/*gal2Δ*) grew at 60% of the specific growth rate observed in the absence of d-glucose (*µ* = 0.057 ± 0.003 h^−1^ versus 0.099 ± 0.001 h^−1^, Fig. 2b). This result indicated that expression of *Pc*AraT in strain IMX869 enabled uptake of l-arabinose in the presence of d-glucose.

### Low residual substrate concentrations in chemostat cultures confirm high-affinity l-arabinose transport kinetics of *Pc*AraT

To further evaluate the in vivo impact of l-arabinose transport via *Pc*AraT, biomass-specific l-arabinose consumption rates and residual substrate concentrations were analysed in l-arabinose-limited, aerobic chemostat cultures, grown at a dilution rate of 0.05 h^−1^. Under these conditions, the l-arabinose-metabolizing strain IMX1508 (*PcaraT/gal2Δ*) exhibited a residual l-arabinose concentration of only 4.2 × 10^−3^ g L^−1^, compared to 1.8 g L^−1^ in cultures of strain IMX929 (*GAL2*) (Table 5). In these growth experiments, different promoters were used for expression of *PcaraT* and *GAL2* (pADH1 and derepressed pGAL2, respectively). However, whilst this may moderately affect expression levels of the two transporters, this cannot explain the over 1000-fold difference in residual l-arabinose concentration. This difference was entirely consistent with the conclusion from the kinetic analyses of ^14^C-l-arabinose uptake, in which both transporter genes were expressed from the same promoter (pHXT7) and which also indicated that *PcaraT* encodes an l-arabinose transporter with a much higher affinity for l-arabinose than Gal2. In shake flask batch cultures grown on an initial l-arabinose concentration of 7.5 g L^−1^, these strains exhibited initial specific growth rates of 0.085 and 0.13 h^−1^, respectively. Based on this observation and on the high *K*_m_ of Gal2 for l-arabinose ([19, 25], this study), the in vivo activity of *Pc*AraT can be expected to exceed that of Gal2 when l-arabinose concentrations are below ca. 4 g L^−1^.

**Table 5:** Physiological data derived from steady-state chemostat cultures of engineered l-arabinose-metabolizing *S. cerevisiae* strains

	IMX929 (*GAL2*)	IMX1508 (*PcaraT*)
Residual l-arabinose (g L^−1^)	1.77 ± 0.19	0.004 ± 0.002
*Y*_X/S_ [g biomass (g l-arabinose)^−1^]	0.48 ± 0.06	0.40 ± 0.01
*q*_l-arabinose_ (mmol g^−1^ h^−1^)	0.70 ± 0.10	0.80 ± 0.08

Strains expressing either *GAL2* (IMX929) or *PcaraT* (IMX1508) as the sole functional l-arabinose transporter were grown in aerobic, l-arabinose-limited chemostat cultures (7.5 g L^−1^ l-arabinose, dilution rate = 0.05 h^−1^, pH 5, *T* = 30 °C). Data are derived from independent triplicate experiments and presented as average ± mean deviation

In duplicate steady-state chemostat cultures, the biomass-specific l-arabinose consumption rate of strain IMX1508 (*PcaraT*) was approximately 14% higher than the one of strain IMX929 (*GAL2*; 0.8 ± 0.1 and 0.7 ± 0.1 mmol g^−1^ h^−1^), reflecting the slightly lower biomass yield of the former strain. This difference in biomass yield is close to the difference of 8.1% that, based on published estimates of the *P*/*O* ratio and proton stoichiometry of the plasma membrane ATPase in aerobic *S. cerevisiae* cultures (both close to 1.0, [63, 64]), would be expected if l-arabinose uptake via *Pc*AraT occurred via symport with a single proton.

## Discussion

Chemostat-based transcriptome analysis of *P. chrysogenum* proved to be an efficient method to identify candidate genes for l-arabinose transporters in this fungus. In comparison with similar studies in batch cultures, use of chemostat cultures offered several advantages. First, chemostat cultivation at a fixed dilution rate eliminated the impact of specific growth rate on transcriptional regulation [65]. Furthermore, use of l-arabinose-limited chemostat cultures of *P. chrysogenum*, in which residual concentrations of this pentose were very low, enabled a focus on the identification of high-affinity transporters. Finally, the use of both d-glucose- and ethanol-limited cultures as references helped to eliminate transcriptional responses of *P. chrysogenum* that were specific to either of these two carbon sources, e.g. as a result of CreA-mediated d-glucose repression of relevant transporter genes [66, 67]. Although this study was focused on l-arabinose transport, the *P. chrysogenum* transcriptome dataset from d-glucose, ethanol and arabinose grown cultures generated in this study [available via GEO, (https://www.ncbi.nlm.nih.gov/geo/) under Accession Numbers GSE12632, GSE24212, and GSE104491, respectively] may contribute to studies on other aspects on metabolism and metabolic regulation in this industrially relevant fungus.

Of five putative transporter genes that showed an over 30-fold higher transcript level in l-arabinose-limited chemostat cultures of *P. chrysogenum* than in d-glucose-limited cultures, only *PcaraT* was shown to encode an l-arabinose transporter that is functional in *S. cerevisiae*. Whilst the low *K*_m_ of this transporter observed upon its expression in *S. cerevisiae* is consistent with its upregulation in l-arabinose-limited cultures of *P. chrysogenum*, this observation does not necessarily imply that *Pc*AraT is the only or even the most important l-arabinose transporter active in these cultures. Problems in protein folding, plasma membrane (mis)targeting, post-translational modification and/or protein turnover [21, 68] may have affected expression of the other candidate genes. Indeed, in screening of cDNA libraries encoding putative heterologous transporters, typically only few of the candidate genes are found to enable transport of the substrate upon expression in *S. cerevisiae* [69, 70].

**Fig. 3:**
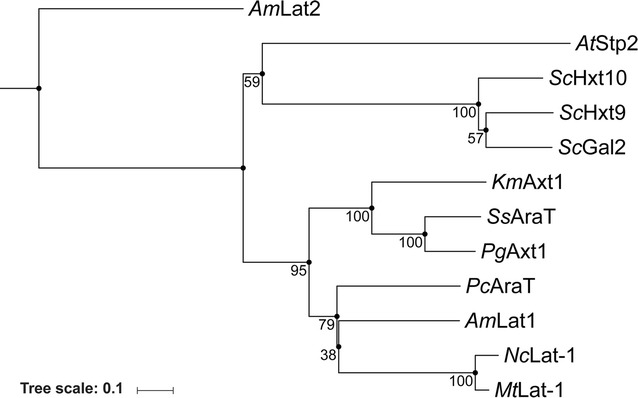
Phylogenetic tree of *S. cerevisiae* Gal2, *Pc*AraT and other heterologous l-arabinose transporters that have previously been functionally expressed in *S. cerevisiae.* Species names are added in two-letter code in front of protein names. Numbers are derived from a 500 times bootstrap iteration. Characteristics and literature references for each transporter are provided in Table 6. Accession numbers: *Sc*Gal2: P13181, *Pc*AraT: CAP85508, *Ss*AraT: A3LQQ5-1, *At*Stp2: OAP13698, *Km*Axt1: GZ791039, *Pg*Axt1: GZ791040, *Am*Lat1: AY923868, *Am*Lat2: AY923869, *Nc*Lat-1: EAA30346, *Mt*Lat-1: G2QFT5-1

Several studies have used *gal2Δ* strains of *S. cerevisiae* to analyse transport kinetics of heterologous l-arabinose transporters (Table 6, Fig. 3). Two studies that estimated *K*_m_ and *V*_max_ of Gal2 upon its reintroduction in such a strain found different results (Table 6) [19, 26]. At l-arabinose concentrations of about 10 mmol L^−1^, these studies reported Gal2-mediated transport rates of 0.3 and 8.9 nmol (mg biomass)^−1^ min^−1^, respectively, as compared to a value of 2.5 nmol (mg biomass)^−1^ min^−1^ observed in the present study. One of the previous studies [26] used a strain that also expressed a functional bacterial l-arabinose pathway, thereby raising the possibility that apparent uptake rates were enhanced by subsequent metabolism of l-arabinose. Moreover, in different studies, *GAL2* was expressed from different promoters (*pTDH3*, *pADH1* and *pHXT7*) and either high-copy-number (2µm) [19, 26] or low-copy-number centromeric (this study) expression plasmids. d-Glucose transport kinetics via Gal2 determined in this study [*K*_m_ = 1.9 mmol L^−1^, *V*_max_ = 26 nmol (mg biomass)^−1^ min^−1^] were similar to previously reported values [1.5 mmol L^−1^ and 27 nmol (mg biomass)^−1^ min^−1^] [20].

**Table 6:** Comparison of key characteristics of Gal2, *Pc*AraT and heterologous l-arabinose transporters that were previously expressed in *S. cerevisiae*

Protein	Origin	*K*_m, ARA_ [mM]	*V*_max, ARA_ [nmol (g biomass)^−1^ min^−1^]	GLC transport	XYL transport	Mechanism	References
*Sc*Gal2	*S. cerevisiae*	335 ± 21.0 57 ± 11 371 ± 19	75 ± 5 2.2 ± 0.3 18 ± 0.8	✓	✓	Facilitated diffusion	This study [19] [26]
*Pc*AraT	*P. chrysogenum*	0.13 ± 0.03	5.3 ± 0.2	✗	✗	H^+^ symport	This study
*Ss*AraT	*Scheffersomyces stipitis*	3.8 ± 1.7	0.4 ± 0.1	✓	✗	nd	[19]
*At*Stp2	*Arabidopsis thaliana*	4.5 ± 2.2	0.6 ± 0.1	✗	✗	H^+^ symport	[19]
*Km*Axt1	*Kluyveromyces marxianus*	263 ± 57	57 ± 6	✗	✓	Facilitated diffusion	[26]
*Pg*Axt1	*Pichia guilliermondii*	0.13 ± 0.04	18 ± 0.8	✗	✓	H^+^ symport	[26]
*Am*Lat1	*Ambrosiozyma monospora*	0.03*	0.2 ± 0.0	✗	✗	nd	[70, 73]
*Am*Lat2	*A. monospora*	nd	4 ± 0	✗	✗	nd	[70, 73]
*Nc*Lat-1	*Neurospora crassa*	58 ± 4	1945 ± 50	✓	nd	H^+^ symport	[30]
*Mt*Lat-1	*Myceliophthora. thermophila*	29 ± 4	172 ± 6	✗	nd	H^+^ symport	[30]

nd, not determined; ARA, l-arabinose; GLC, d-glucose; XYL, d-xylose. * *K*_m_ of *Am*Lat1 was determined as a GFP-fusion protein [73]

l-Arabinose transport rates in l-arabinose-limited chemostat cultures of both Gal2- and *Pc*AraT-dependent strains were higher than the *V*_max_ values calculated from transporter assays with radioactively labelled l-arabinose. A similar difference between transport assays and rates of l-arabinose uptake in growing cultures was reported by Knoshaug et al. [26]. These discrepancies suggest that either the transport assays did not accurately reflect zero-trans-influx kinetics [71] or that differences in experimental conditions and/or cellular energy status between transport assays and chemostat cultures influenced l-arabinose uptake. Assuming that *Pc*AraT mediates symport of l-arabinose with a single proton, the l-arabinose consumption rate in aerobic, l-arabinose-limited chemostat cultures of the strain IMX1508 (*Pc*AraT *gal2Δ*) (0.8 mmol (g biomass)^−1^ h^−1^; Table 5) would, under anaerobic conditions, correspond to an ATP production rate of ca. 0.3 mmol ATP (g biomass)^−1^ h^−1^. This rate of ATP production is well below the reported ATP requirement of anaerobic *S. cerevisiae* cultures for cellular maintenance [ca. 1 mmol ATP (g biomass)^−1^ h^−1^] [72]. Consistent with this observation, no growth on l-arabinose as the sole carbon source was observed in anaerobic shake flask cultures of the l-arabinose specialist strain IMX869 strain (*Pc*AraT *gal2Δ*) (data not shown).

Differences in experimental protocols for strain construction and sugar uptake studies, as well as the different kinetics observed in transport assays and growing cultures, complicate quantitative comparisons between different studies. Nevertheless, some important differences can be discerned between the heterologous l-arabinose transporters that have hitherto been expressed in *S. cerevisiae* (Table 6, Fig. 3). Protein sequence alignment of *Pc*AraT and transporters that were previously shown to mediate l-arabinose import in *S. cerevisiae* showed that *Pc*AraT clusters with *Ambrosiozyma monospora Am*Lat1 (Fig. 3). In terms of its low *K*_m_, *Pc*AraT most closely resembled *Am*Lat1 and the *P. guilliermondii Pg*Axt1 transporter. However, expression in *S. cerevisiae* of *Am*Lat1 [70, 73] led to ~ 25-fold lower reported *V*_max_ of l-arabinose uptake than found in the present study for *Pc*AraT. In contrast to *Pc*AraT, *Pg*Axt was able to transport d-glucose, which might contribute to the strong inhibition of the latter transporter by d-glucose [26]. Although *Pc*AraT resembled *A. thaliana* Stp2 [19] in being partially inhibited by d-glucose despite an inability to transport this sugar, *Pc*AraT enabled consumption of l-arabinose in batch cultures containing 20 g L^−1^ d-glucose.

In common with other high-affinity sugar transporters in yeasts and fungi [26, 74], the observation that *Pc*AraT mediates l-arabinose-proton symport should be taken into account in future strain designs, since simultaneous activity of proton symport and facilitated diffusion, e.g. via Gal2, may result in energy-consuming futile cycles [8].

In the lignocellulosic hydrolysates now used in the first industrial-scale plants for ‘second generation’ bioethanol production, l-arabinose generally represents between 2 and 3% of the total sugars [8]. At the resulting low concentrations of l-arabinose in the industrial processes, Gal2 operates far from substrate saturation and is, moreover, strongly inhibited by d-glucose. Based on its kinetic characteristics, as analysed in transport assays and growing cultures, *Pc*AraT represents an interesting candidate transporter for evaluation of l-arabinose co-consumption under industrial conditions. If the characteristics of *Pc*AraT determined in the present study can be reproduced in industrial strains and under industrial conditions, this transporter can contribute to a timely and efficient conversion of l-arabinose, and thereby to the overall process economics.

## Conclusion

Transcriptome analyses of l-arabinose-limited *P. chrysogenum* chemostat cultures proved valuable for identification of the high-affinity l-arabinose transporter *Pc*AraT. Functional expression and characterization in *S. cerevisiae* revealed a high affinity and specificity of this transporter for l-arabinose (*K*_m_ = 0.13 mmol L^−1^), combined with a limited sensitivity to inhibition by d-glucose and d-xylose, which are present at high concentrations in lignocellulosic hydrolysates. These characteristics differentiate *Pc*AraT from the endogenous *S. cerevisiae* transporter capable of l-arabinose transport (Gal2) and qualify it as a potentially valuable additional element in metabolic engineering strategies towards efficient and complete conversion of l-arabinose present in second-generation feedstocks for yeast-based production of fuels and chemicals.

## Additional files

**Additional file 1.** Primers used in this study.

**Additional file 2.** Differentially expressed genes in *P. chrysogenum* chemostat cultures over three different conditions (d-glucose-, l-arabinose, and ethanol-limited). The table includes 540 genes that were differentially expressed amongst d-glucose-, l-arabinose, and ethanol-limited chemostat cultures of *P. chrysogenum* (7.5 g L^−1^, 7.5 g L^−1^, 5.8 g L^−1^, respectively, D = 0.03 h^−1^, T = 30 °C). 137 transcripts exhibited a fold-change higher than three on l-arabinose and lower than three on ethanol relative to the d-glucose condition (the top 137 genes presented in the table). Shown are averages from triplicate (d-glucose) or duplicate chemostat cultures (l-arabinose, ethanol), the respective standard deviation and the ratio of gene expression levels in the presence of l-arabinose over d-glucose (Ara vs. Glc), as well as ethanol over d-glucose (EtOH vs. Glc).

**Additional file 3.** Effect of d-glucose (**a**) and d-xylose (**b**) on the specific rate of l-arabinose uptake by *Pc*AraT (filled triangles) and Gal2 (filled squares). Uptake experiments were performed with 50 mmol L^−1^ [^14^C-] l-arabinose in the presence of increasing concentrations of d-glucose (**a**) or d-xylose (**b**). Symbols indicate uptake rates observed with the Hxt1-7 and Gal2 deletion strain *S. cerevisiae* DS68625-*PcaraT* (filled triangles) and DS68625-*GAL2* (filled squares), expressing either *Pc*AraT or Gal2, respectively. Data are derived from duplicate experiments and shown as the average ± mean deviation.

**Additional file 4.** Specific rate of l-arabinose uptake by *Pc*AraT. Uptake experiments were performed with increasing concentrations of [^14^C-] l-arabinose with the Hxt1-7 and Gal2 deletion strain *S. cerevisiae* DS68625-*PcaraT* expressing *PcaraT* on a centromeric plasmid. No [^14^C-] d-glucose uptake was observed for this strain. Data are derived from duplicate experiments and shown as the average ± mean deviation.

**Additional file 5.** Specific rate of d-glucose uptake by Gal2. Uptake experiments were performed with increasing concentrations of [^14^C-] d-glucose with the Hxt1-7 and Gal2 deletion strain *S. cerevisiae* DS68625-*GAL2* expressing *GAL2* on a centromeric plasmid. Data are derived from duplicate experiments and shown as the average ± mean deviation.

**Additional file 6.** Specific rate of l-arabinose uptake by Gal2. Uptake experiments were performed with increasing concentrations of [^14^C-] l-arabinose with the Hxt1-7 and Gal2 deletion strain *S. cerevisiae* DS68625-*GAL2* expressing *GAL2* on a centromeric plasmid. Data are derived from duplicate experiments and shown as the average ± mean deviation.

**Additional file 7.** Impact of proton-gradient uncoupling on transport activity. Transport rates of [^14^C]-l-arabinose of the Hxt1-7 and Gal2 deletion strains DS68625-*PcaraT* and DS68625-*GAL2* expressing either *Pc*AraT (DS68625-*PcaraT*, white bars) or Gal2 (DS68625-*GAL2*, grey bars) on a centromeric plasmid. Transport rates were determined in 200 μl synthetic medium at a [^14^C]-l-arabinose concentration of 2 mmol L^−1^ upon addition of either 0.5 µL water, 0.5 μL DMSO, or 10 μM CCCP (0.5 µl of a stock solution dissolved in 100% DMSO) (A). Panel (B) shows the uptake capacity in % relative to the control (H_2_O). Data are derived from duplicate experiments and shown as the average ± mean deviation.

## Abbreviations

SM: synthetic medium
SMA: synthetic medium with 20 g L^−1^ l-arabinose
SMAG: synthetic medium with 20 g L^−1^ l-arabinose and 20 g L^−1^ d-glucose
SMEG: synthetic medium with 2% ethanol and 3% glycerol
SM-urea: synthetic medium with urea
YPD: yeast peptone medium with 20 g L^−1^ d-glucose
YPA: yeast peptone medium with 20 g L^−1^ l-arabinose
YPEG: yeast peptone medium with 2% ethanol and 3% glycerol
ARA: l-arabinose
GLC: d-glucose
XYL: d-xylose
OD: optical density
ORF: open-reading frame
nd: not determined
PPP: pentose phosphate pathway
