# Supplementary material for: The Penicillium chrysogenum transporter PcAraT enables high-affinity, glucose-insensitive l-arabinose transport in Saccharomyces cerevisiae
Source: Biotechnol Biofuels. 2018 Mar 13;11:63. doi: 10.1186/s13068-018-1047-6 (PMC5848512; doi:10.1186/s13068-018-1047-6)
Supplement: Supplementary file 1 — Additional file 1. Primers used in this study. [file 13068_2018_1047_MOESM1_ESM.docx]

Additional File 1

| **Primers used for amplification of integration fragments:** | | | | | | |
| --- | --- | --- | --- | --- | --- | --- |
| Primer nr.: | Purpose: | Template: | | | Sequence 5’ -> 3’: | |
| 4653 | flGal1*-cas9-A* fragment | p414-TEF1p-cas9-CYC1t | | | GTGCCTATTGATGATCTGGCGGAATGTCTGCCGTGCCATAGCCATGCCTTCACATATAGTCCGCAAATTAAAGCCTTCGAG | |
| 5981 | flGal1*-cas9-A* fragment | p414-TEF1p-cas9-CYC1t | | | TTCACCGGTCGCGTTCCTGAAACGCAGATGTGCCTCGCGCCGCACACCGTATTACCGCCTTTG | |
| 3093 | A-AmdSYM-flGal80 fragment | pUG-amdSYM | | | ACTATATGTGAAGGCATGGCTATGGCACGGCAGACATTCCGCCAGATCATCAATAGGCACCTTCGTACGCTGCAGGTCGAC | |
| 1678 | A-AmdSYM-flGal80 fragment | pUG-amdSYM | | | AATGAGAAGTTGTTCTGAACAAAGTAAAAAAAAGAAGTATACTTACATAGGCCACTAGTGGATCTG | |
| 5910 | flGRE3_G fragment | CEN.PK113-7D | | | CCACCTGGTGGAACATCCTAGAAC | |
| 5911 | flGRE3_G fragment | CEN.PK113-7D | | | AAGGGCCATGACCACCTGATGCACCAATTAGGTAGGTCTGGCTATGTCTATACCTCTGGCCTACCAGCAACAATTGGGAAAC | |
| 7133 | fl_*RPE1*_H fragment | pUD347 | | | TATAATATTTCATTATCGGAACTCTAGATTCTATACTTGTTTCCCAATTGTTGCTGGTAGGGCCCTTCCGGGAGTTTATC | |
| 3290 | fl_*RPE1*_H fragment | pUD347 | | | GTCACGGGTTCTCAGCAATTCG | |
| 3291 | H_*TKL1*_I fragment | pUD348 | | | CTCTAACGCCTCAGCCATCATCG | |
| 4068 | H_*TKL1*_I fragment | pUD348 | | | GCCTACGGTTCCCGAAGTATGC | |
| 3274 | I_*TAL1*_A fragment | pUD349 | | | TATTCACGTAGACGGATAGGTATAGC | |
| 3275 | I_*TAL1*_A fragment | pUD349 | | | GTGCCTATTGATGATCTGGCGGAATG | |
| 3847 | A_ *NQM1* _B fragment | pUD344 | | | ACTATATGTGAAGGCATGGCTATGG | |
| 3276 | A_ *NQM1*_B fragment | pUD344 | | | GTTGAACATTCTTAGGCTGGTCGAATC | |
| 4672 | B_*RKI1*_C fragment | pUD345 | | | CACCTTTCGAGAGGACGATG | |
| 3277 | B_*RKI1*_C fragment | pUD345 | | | CTAGCGTGTCCTCGCATAGTTCTTAGATTG | |
| 3283 | C_*TKL2*_F fragment | pUD346 | | | ACGTCTCACGGATCGTATATGC | |
| 3288 | C_*TKL2*_F fragment | pUD346 | | | TGCCGAACTTTCCCTGTATGAAGC | |
| 5936 | F_flGRE3 | CEN.PK113-7D | | | CATACGTTGAAACTACGGCAAAGGATTGGTCAGATCGCTTCATACAGGGAAAGTTCGGCACCCTCATTCCGATGCTGTATATGTG | |
| 5937 | F_flGRE3 | CEN.PK113-7D | | | ACTGCTTCGTCTAGGTCTTG | |
| 6628 | flGal80_*AraA*_G fragment | pUD354 | | | TCCTTGCCGACCAGCGTATACAATCTCGATAGTTGGTTTCCCGTTCTTTCCACTCCCGTCCGCGCAGATTAGCGAAGC | |
| 6285 | flGal80_*AraA*_G fragment | pUD354 | | | AAGGGCCATGACCACCTGATGCACCAATTAGGTAGGTCTGGCTATGTCTATACCTCTGGCGCGATACCCTGCGATCTTC | |
| 6273 | G_*AraA*_A fragment | pUD354 | | | GCCAGAGGTATAGACATAGCCAGACCTACCTAATTGGTGCATCAGGTGGTCATGGCCCTTCGCGCAGATTAGCGAAGC | |
| 6280 | G_*AraA*_A fragment | pUD354 | | | GTGCCTATTGATGATCTGGCGGAATGTCTGCCGTGCCATAGCCATGCCTTCACATATAGTGCGATACCCTGCGATCTTC | |
| 6270 | A_*AraA*_B fragment | pUD354 | | | ACTATATGTGAAGGCATGGCTATGGCACGGCAGACATTCCGCCAGATCATCAATAGGCACCGCGCAGATTAGCGAAGC | |
| 6281 | A_*AraA*_B fragment | pUD354 | | | GTTGAACATTCTTAGGCTGGTCGAATCATTTAGACACGGGCATCGTCCTCTCGAAAGGTGGCGATACCCTGCGATCTTC | |
| 6271 | B_*AraA*_C fragment | pUD354 | | | CACCTTTCGAGAGGACGATGCCCGTGTCTAAATGATTCGACCAGCCTAAGAATGTTCAACCGCGCAGATTAGCGAAGC | |
| 6282 | B_*AraA*_C fragment | pUD354 | | | CTAGCGTGTCCTCGCATAGTTCTTAGATTGTCGCTACGGCATATACGATCCGTGAGACGTGCGATACCCTGCGATCTTC | |
| 6272 | C_*AraA*_D fragment | pUD354 | | | ACGTCTCACGGATCGTATATGCCGTAGCGACAATCTAAGAACTATGCGAGGACACGCTAGCGCGCAGATTAGCGAAGC | |
| 6284 | C_*AraA*_D fragment | pUD354 | | | AATCACTCTCCATACAGGGTTTCATACATTTCTCCACGGGACCCACAGTCGTAGATGCGTGCGATACCCTGCGATCTTC | |
| 6283 | D_*AraA*_M fragment | pUD354 | | | ACGCATCTACGACTGTGGGTCCCGTGGAGAAATGTATGAAACCCTGTATGGAGAGTGATTGCGATACCCTGCGATCTTC | |
| 6275 | D_*AraA*_M fragment | pUD354 | | | ACGAGAGATGAAGGCTCACCGATGGACTTAGTATGATGCCATGCTGGAAGCTCCGGTCATCGCGCAGATTAGCGAAGC | |
| 6287 | M_*AraA*_N fragment | pUD354 | | | ATGACCGGAGCTTCCAGCATGGCATCATACTAAGTCCATCGGTGAGCCTTCATCTCTCGTGCGATACCCTGCGATCTTC | |
| 6276 | M_*AraA*_N fragment | pUD354 | | | TTCTAGGCTTTGATGCAAGGTCCACATATCTTCGTTAGGACTCAATCGTGGCTGCTGATCCGCGCAGATTAGCGAAGC | |
| 6288 | N_*AraA*_O fragment | pUD354 | | | GATCAGCAGCCACGATTGAGTCCTAACGAAGATATGTGGACCTTGCATCAAAGCCTAGAAGCGATACCCTGCGATCTTC | |
| 6277 | N_*AraA*_O fragment | pUD354 | | | ATACTCCCTGCACAGATGAGTCAAGCTATTGAACACCGAGAACGCGCTGAACGATCATTCCGCGCAGATTAGCGAAGC | |
| 6289 | O_*AraA*_I fragment | pUD354 | | | GAATGATCGTTCAGCGCGTTCTCGGTGTTCAATAGCTTGACTCATCTGTGCAGGGAGTATGCGATACCCTGCGATCTTC | |
| 6274 | O_*AraA*_I fragment | pUD354 | | | GCCTACGGTTCCCGAAGTATGCTGCTGATGTCTGGCTATACCTATCCGTCTACGTGAATACGCGCAGATTAGCGAAGC | |
| 3274 | I_*AraB_*K fragment | pUD355 | | | TATTCACGTAGACGGATAGGTATAGC | |
| 6636 | I_*AraB_*K fragment | pUD355 | | | GCGAGGACTTCCCATCAATTGC | |
| 6634 | K_*AraD_*flGal80 | pUD356 | | | AAGATAGTCGCCGAACTCGC | |
| 6635 | K_*AraD_*flGal80 | pUD356 | | | CTCAGTATTCGTTTTTATAACGTTCGCTGCACTGGGGGCCAAGCACAGGGCAAGATGCTTTGCCGAACTTTCCCTGTATG | |
| 7676 | *PcaraT* fragment | pPWT118 | | | TTTCTAATGCCTTTTCCATCATGTTACTACGAGTTTTCTGAACCTCCTCGCACATTGGTATCTTCACGCGTGTTCGAG | |
| 7660 | *PcaraT* fragment | pPWT118 | | | TATAAATATTTATCGTCACGAATAAATCCCGTGAATTTCTAACAAAGTTTATACAATATCTAACCTCGGAAGATCGTCGACAAG | |
| 2641 | Ura3 repair fragment | CEN.PK113-7D | | | ATTGCCCAGTATTCTTAACC | |
| 1522 | Ura3 repair fragment | CEN.PK113-7D | | | CGAGATTCCCGGGTAATAACTG | |
| 2788 | *HXK2* KO cassette | pUG-72 | | | ATTGTAGGAATATAATTCTCCACACATAATAAGTACGTTAATATAAACAGCTGAAGCTTCGTACGC | |
| 2789 | *HXK2* KO cassette | pUG-72 | | | TTAAAAAAAGGGCACCTTCTTGTTGTTCAAACTTAATTTACAAATTAAGTGCATAGGCCACTAGTGGATCTG | |
| 10585 | flGal2_*Pc13g04640_*flGal2 | pPWT111 | | | ACACAAATAATAGGTTTAGGTAAGGAATTTATATAATCGTAAGGATATCATTGATAAGGAGTTTATCATTATCAATACTGCCATTTCAAAG | |
| 10584 | flGal2_*Pc13g04640_*flGal2 | pPWT111 | | | TTAGTTTTGTAGACATATATAAACAATCAGTAATTGGATTGAAAATTTGGTGTTGTGAATTAACCTCGGAAGATCGTCGACAAG | |
| 10585 | flGal2_*Pc13g08230_*flGal2 | pPWT113 | | | ACACAAATAATAGGTTTAGGTAAGGAATTTATATAATCGTAAGGATATCATTGATAAGGAGTTTATCATTATCAATACTGCCATTTCAAAG | |
| 10584 | flGal2_*Pc13g08230_*flGal2 | pPWT113 | | | TTAGTTTTGTAGACATATATAAACAATCAGTAATTGGATTGAAAATTTGGTGTTGTGAATTAACCTCGGAAGATCGTCGACAAG | |
| 10585 | flGal2_*Pc16g05670_*flGal2 | pPWT116 | | | ACACAAATAATAGGTTTAGGTAAGGAATTTATATAATCGTAAGGATATCATTGATAAGGAGTTTATCATTATCAATACTGCCATTTCAAAG | |
| 10584 | flGal2_*Pc16g05670_*flGal2 | pPWT116 | | | TTAGTTTTGTAGACATATATAAACAATCAGTAATTGGATTGAAAATTTGGTGTTGTGAATTAACCTCGGAAGATCGTCGACAAG | |
| 10585 | flGal2*_PcaraT_*flGal2*_*flGal2 | pPWT118 | | | ACACAAATAATAGGTTTAGGTAAGGAATTTATATAATCGTAAGGATATCATTGATAAGGAGTTTATCATTATCAATACTGCCATTTCAAAG | |
| 10584 | flGal2*_PcaraT_*flGal2*_*flGal2 | pPWT118 | | | TTAGTTTTGTAGACATATATAAACAATCAGTAATTGGATTGAAAATTTGGTGTTGTGAATTAACCTCGGAAGATCGTCGACAAG | |
| 10585 | flGal2_*Pc22g14520_*flGal2 | pPWT123 | | | ACACAAATAATAGGTTTAGGTAAGGAATTTATATAATCGTAAGGATATCATTGATAAGGAGTTTATCATTATCAATACTGCCATTTCAAAG | |
| 10584 | flGal2_*Pc22g14520_*flGal2 | pPWT123 | | | TTAGTTTTGTAGACATATATAAACAATCAGTAATTGGATTGAAAATTTGGTGTTGTGAATTAACCTCGGAAGATCGTCGACAAG | |
| 9563 | *GAL2* KO cassette | - | | | GGATTGAAAATTTGGTGTTGTGAATTGCTCTTCATTATGCACCTTATTCAATTATCATCAGATAACATGCTCTGCCATCCTTTGTTCACCGAGCAAAATTAAAAACGCAAAATGAATTGT | |
| 9564 | *GAL2* KO cassette | - | | | ACAATTCATTTTGCGTTTTTAATTTTGCTCGGTGAACAAAGGATGGCAGAGCATGTTATCTGATGATAATTGAATAAGGTGCATAATGAAGAGCAATTCACAACACCAAATTTTCAATCC | |
| 943 | flGal2_*KanMX*_flGal2 | pUG6 | | | TAAGTAAACACAAGATTAACATAATAAAAAAAATAATTCTTTCATAGCATAGGCCACTAGTGGATCTG | |
| 944 | flGal2_Kan*M*X_flGal2 | pUG6 | | | TAAGAGAGATGATGGAGCGTCTCACTTCAAACGCATTATTCCAGCTGAAGCTTCGTACGC | |
| **Primers used for the construction of gRNA expression plasmids:** | | | | | | |
| Primer nr.: | Purpose: | | Template: | Primer nr.: | |  |
| 5792 | pUDE348/335/327 backbone | | pMEL10 | GTTTTAGAGCTAGAAATAGCAAGTTAAAATAAG | |  |
| 5980 | pUDE348/335/327 backbone | | pMEL10 | CGACCGAGTTGCTCTTG | |  |
| 6631 | pUDE348 gRNA | | pMEL10 | ATTTTAACTTGCTATTTCTAGCTCTAAAACAAGATCTCTTGTTGTAGTCCGATCATTTATCTTTCACTGCGG | |  |
| 5979 | pUDE335 gRNA | | pMEL10 | TATTGACGCCGGGCAAGAGC | |  |
| 9283 | pUDR245 gRNA | | pROS10 | TGCGCATGTTTCGGCGTTCGAAACTTCTCCGCAGTGAAAGATAAATGATCGTTGACTACACCAATGACAAGTTTTAGAGCTAGAAATAGCAAGT | |  |
| 9283 | pUDR246 gRNA1 | | pROS10 | TGCGCATGTTTCGGCGTTCGAAACTTCTCCGCAGTGAAAGATAAATGATCGTTGACTACACCAATGACAAGTTTTAGAGCTAGAAATAGCAAGT | |  |
| 10024 | pUDR246 gRNA2 | | pROS10 | TGCGCATGTTTCGGCGTTCGAAACTTCTCCGCAGTGAAAGATAAATGATCTTCATGTTTGGCTGGGATACGTTTTAGAGCTAGAAATAGCAAGTTAAAATAAGGCTAGTCCGTTATCAAC | |  |
| 6005 | pUDR246/245 backbone | | pROS10 | GATCATTTATCTTTCACTGCGGAGAAG | |  |
| 2528 | PCR verification of gRNA | | pUDE335 | TCTTTCCTGCGTTATCCC | |  |
| 960 | PCR verification of gRNA | | pUDE335 | GTGGATGATGTGGTCTCTAC | |  |
| **Primers used for verifying integration of fragments:** | | | | | |  |
| Primer nr.: | Purpose: | | Primer nr.: | | |  |
| 970 | Checking *AraA* integration | | CATTTACCGGCGCACTCTCG | | |  |
| 6925 | Checking *AraA* integration | | GGTGCTTTGGAATGGATG | | |  |
| 6924 | Checking *AraA* integration | | TGTTGAGAACCGGTAACG | | |  |
| 4692 | Checking *AraA* integration | | AAGGGCCATGACCACCTG | | |  |
| 3275 | Checking *AraA* integration | | GTGCCTATTGATGATCTGGCGGAATG | | |  |
| 4173 | Checking *AraA* integration | | GTTGAACATTCTTAGGCTGG | | |  |
| 3277 | Checking *AraA* integration | | CTAGCGTGTCCTCGCATAGTTCTTAGATTG | | |  |
| 5231 | Checking *AraA* integration | | AATCACTCTCCATACAGGG | | |  |
| 3354 | Checking *AraA* integration | | ACGCATCTACGACTGTGGGTC | | |  |
| 4184 | Checking *AraA* integration | | ATGACCGGAGCTTCCAGCATG | | |  |
| 3843 | Checking *AraA* integration | | GATCAGCAGCCACGATTG | | |  |
| 3837 | Checking *AraA* integration | | GAATGATCGTTCAGCGCG | | |  |
| 4068 | Checking *AraA* integration | | GCCTACGGTTCCCGAAGTATGC | | |  |
| 6926 | Checking *AraB* integration | | TGTCTACCGCTGGTGAAGGTG | | |  |
| 6636 | Checking *AraB* integration | | GCGAGGACTTCCCATCAATTGC | | |  |
| 6928 | Checking *AraD* integration | | GAGAAAGCACGGTGCTTCTG | | |  |
| 971 | Checking *AraD* integration | | ATAAGAACACCCGCATGCAC | | |  |
| 1977 | Checking PPP integration | | TACCTTCTGCTCTCTCTG | | |  |
| 5164 | Checking PPP integration | | AAAGGATTCGGGCCCAAATCGG | | |  |
| 3225 | Checking PPP integration | | CTGTGATCTCCAGAGCAAAG | | |  |
| 2673 | Checking PPP integration | | TGAAGTGGTACGGCGATGC | | |  |
| 3878 | Checking PPP integration | | gcgGGTACCCGCCTCGTTTCTTTTTCTTC | | |  |
| 2913 | Checking PPP integration | | AATAGCCGCCAGGAAATGCC | | |  |
| 1999 | Checking PPP integration | | CGCGCTCAACCTGGAATTAC | | |  |
| 2374 | Checking PPP integration | | GCAGAAGTGTCTGAATGTATTAAGG | | |  |
| 3515 | Checking PPP integration | | CTGACAGGTGGTTTGTTACG | | |  |
| 5603 | Checking PPP integration | | CGCAAGTTTATCAATGTCGG | | |  |
| 3927 | Checking PPP integration | | AAGAGAATGGACCTATGAACTGATG | | |  |
| 5396 | Checking PPP integration | | CGAATAAACACACATAAACAAACAAAATGGCACAGTTCTCCGACATTG | | |  |
| 5937 | Checking PPP integration | | ACTGCTTCGTCTAGGTCTTG | | |  |
| 5910 | Checking PPP integration | | CCACCTGGTGGAACATCCTAGAAC | | |  |
| 4657 | Checking PcAraT integration | | TTGCGCTAAGAGAATGGACC | | |  |
| 5905 | Checking PcAraT integration | | CTTTTTTTTAGTTTTAAAACACCAAGAACTTAG | | |  |
| 4930 | Checking Hxk2 deletion | | GGCAAGAGTATAGCGTGATACC | | |  |
| 3070 | Checking Hxk2 deletion | | AGTGCTTCCGTTCGTTCCAG | | |  |
| 3564 | Checking Hxk2 deletion | | TTGGTGCTAGAGCTGCTAGATTG | | |  |
| 2926 | Checking Hxk2 deletion | | ATCAATTCCTTTGGCACATCGGC | | |  |
| 8883 | Checking Gal2 deletion | | AGTTAAGCCCTTCCCATCTC | | |  |
| 8889 | Checking Gal2 deletion | | GCGAAACATAGCCCTAATGG | | |  |
| 8883 | Checking integration of *gal2::PcAraT/ Pc13g04640/ Pc13g08230/ Pc16g05670 Pc22g14520* | | AGTTAAGCCCTTCCCATCTC | | |  |
| 8889 | Checking integration of *gal2::PcAraT/ Pc13g04640/ Pc13g08230/ Pc16g05670 Pc22g14520* | | GCGAAACATAGCCCTAATGG | | |  |
