# Supplementary figures and images for: The Penicillium chrysogenum transporter PcAraT enables high-affinity, glucose-insensitive l-arabinose transport in Saccharomyces cerevisiae
Source: Biotechnol Biofuels. 2018 Mar 13;11:63. doi: 10.1186/s13068-018-1047-6 (PMC5848512; doi:10.1186/s13068-018-1047-6)

Additional File 3

**a**


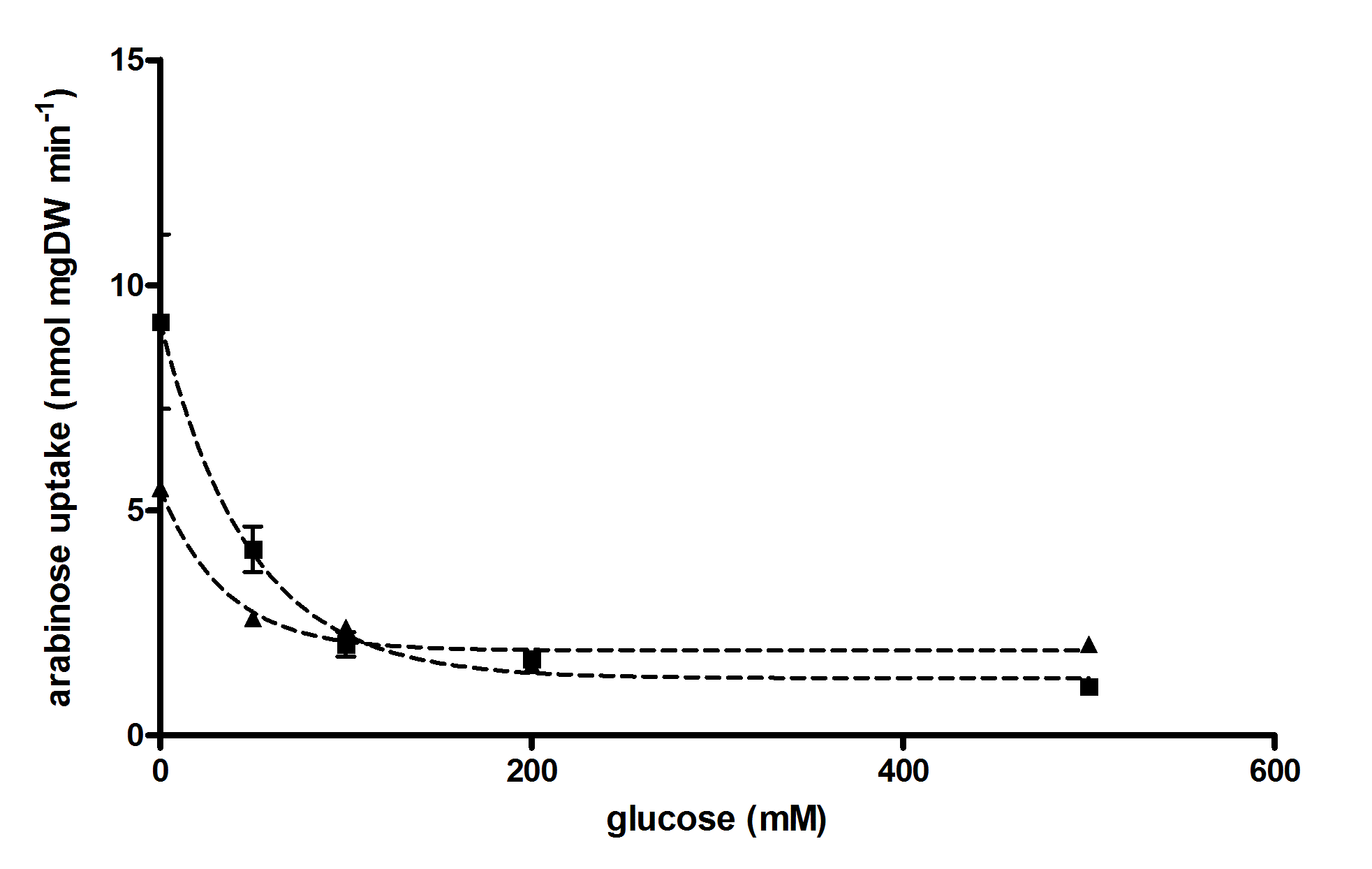


**b**


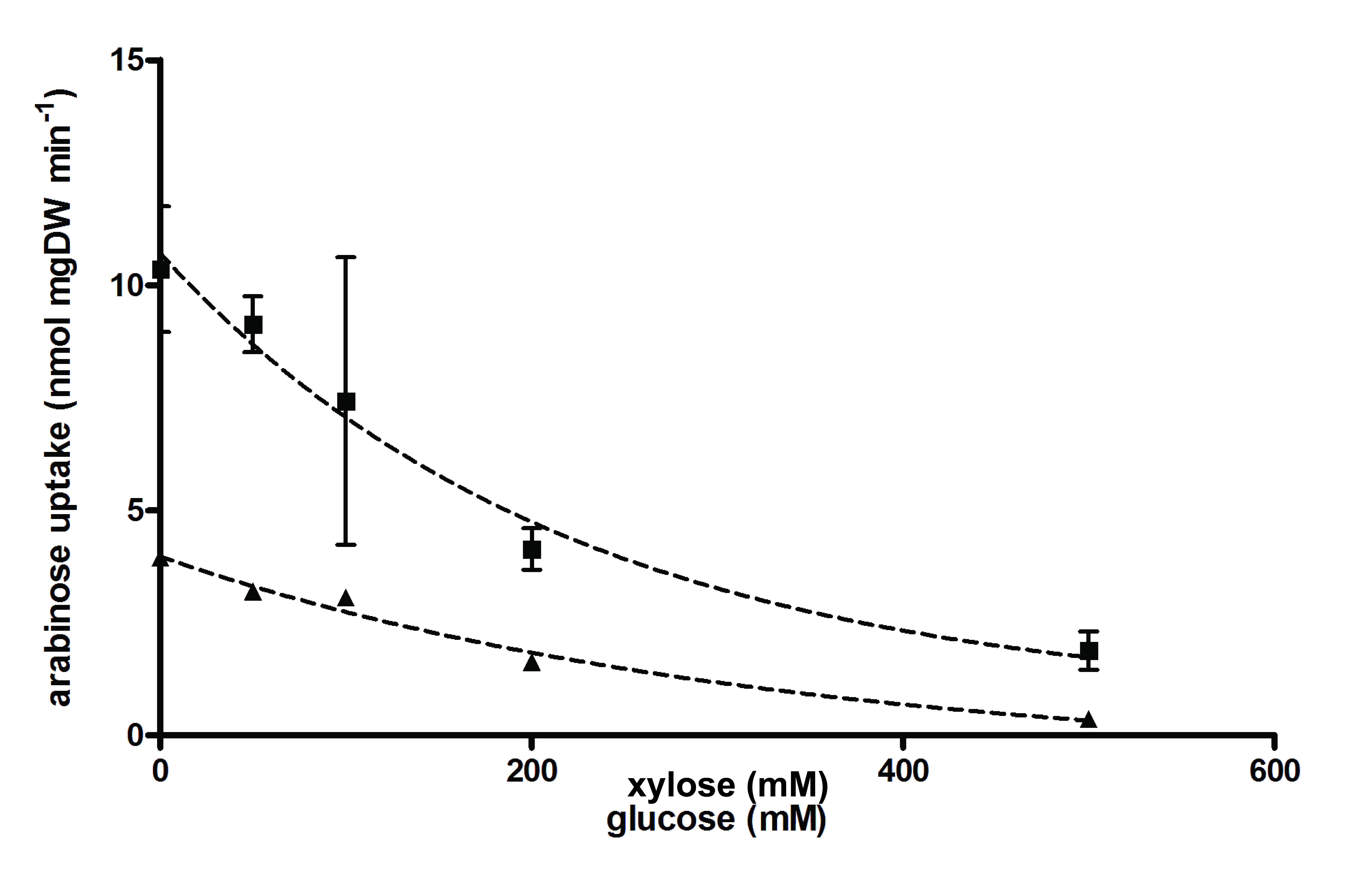

Supplement: Supplementary file 3 — Additional file 3. Effect of d-glucose (a) and d-xylose (b) on the specific rate of l-arabinose uptake by PcAraT (filled triangles) and Gal2 (filled squares). Uptake experiments were performed with 50 mmol L−1 [14C-] l-arabinose in the presence of increasing concentrations of d-glucose (a) or d-xylose (b). Symbols indicate uptake rates observed with the Hxt1-7 and Gal2 deletion strain S. cerevisiae DS68625-PcaraT (filled triangles) and DS68625-GAL2 (filled squares), expressing either PcAraT or Gal2, respectively. Data are derived from duplicate experiments and shown as the average ± mean deviation. [file 13068_2018_1047_MOESM3_ESM.docx]

Additional File 4


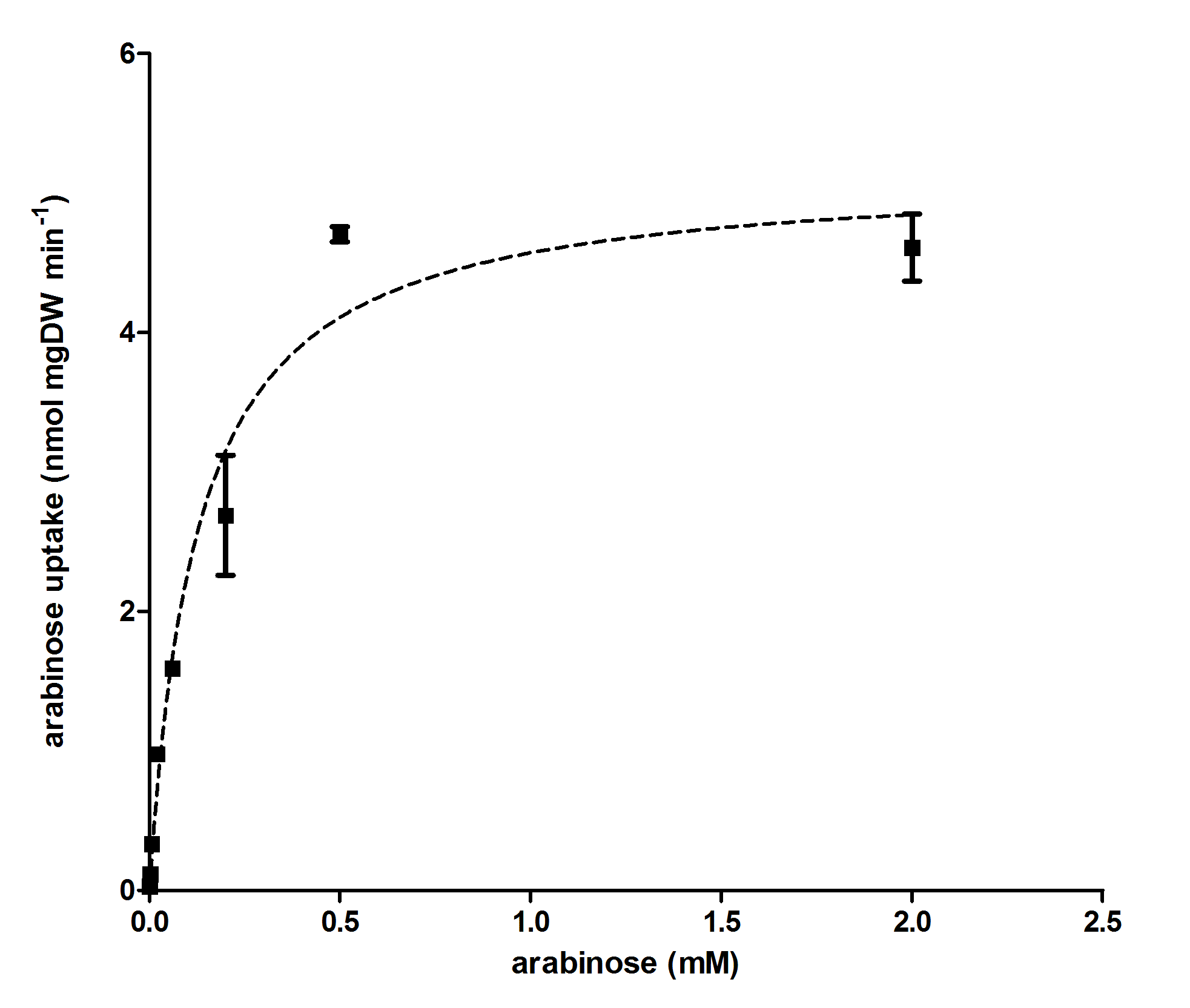

Supplement: Supplementary file 4 — Additional file 4. Specific rate of l-arabinose uptake by PcAraT. Uptake experiments were performed with increasing concentrations of [14C-] l-arabinose with the Hxt1-7 and Gal2 deletion strain S. cerevisiae DS68625-PcaraT expressing PcaraT on a centromeric plasmid. No [14C-] d-glucose uptake was observed for this strain. Data are derived from duplicate experiments and shown as the average ± mean deviation. [file 13068_2018_1047_MOESM4_ESM.docx]

Additional File 5


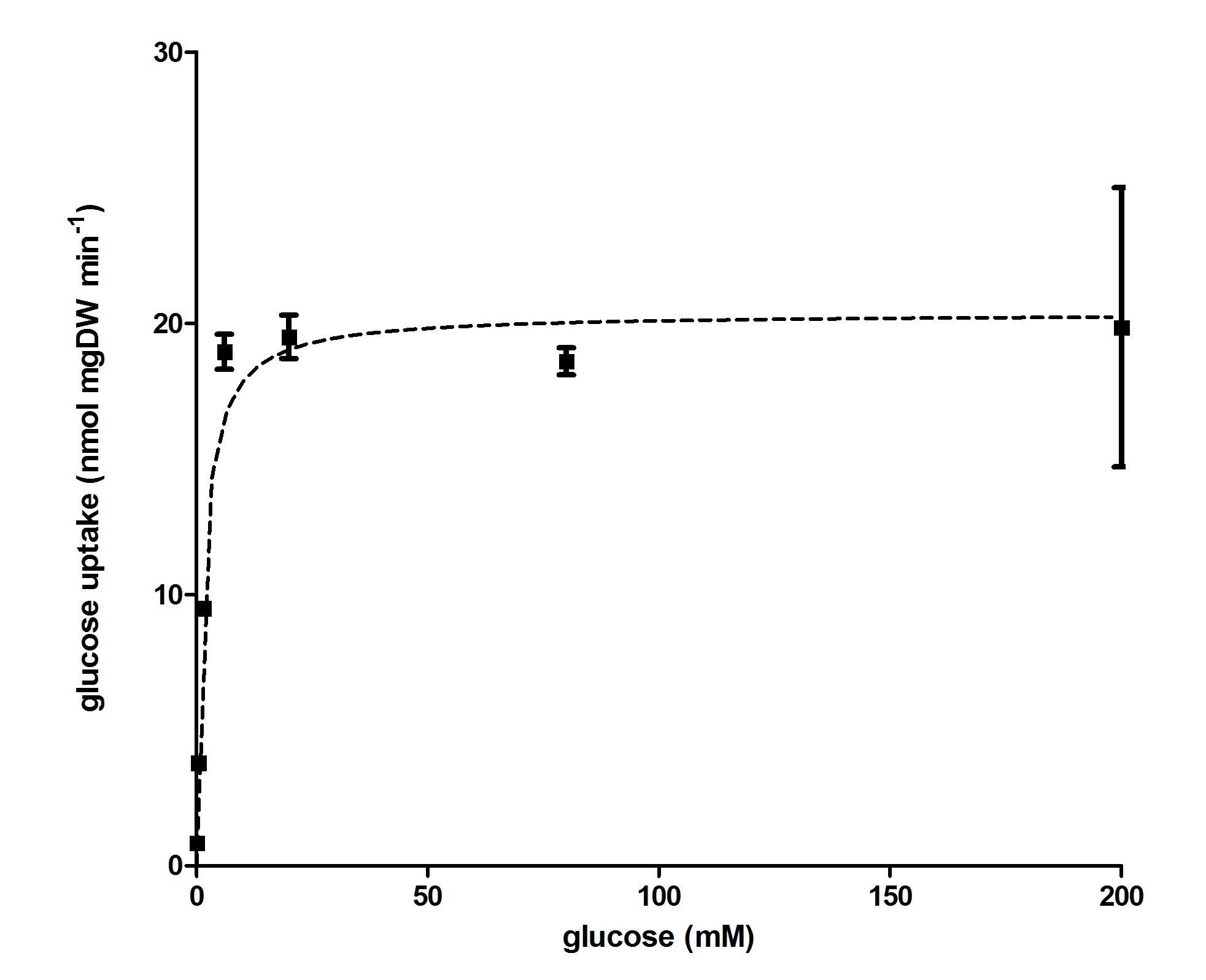

Supplement: Supplementary file 5 — Additional file 5. Specific rate of d-glucose uptake by Gal2. Uptake experiments were performed with increasing concentrations of [14C-] d-glucose with the Hxt1-7 and Gal2 deletion strain S. cerevisiae DS68625-GAL2 expressing GAL2 on a centromeric plasmid. Data are derived from duplicate experiments and shown as the average ± mean deviation. [file 13068_2018_1047_MOESM5_ESM.docx]

Additional File 6


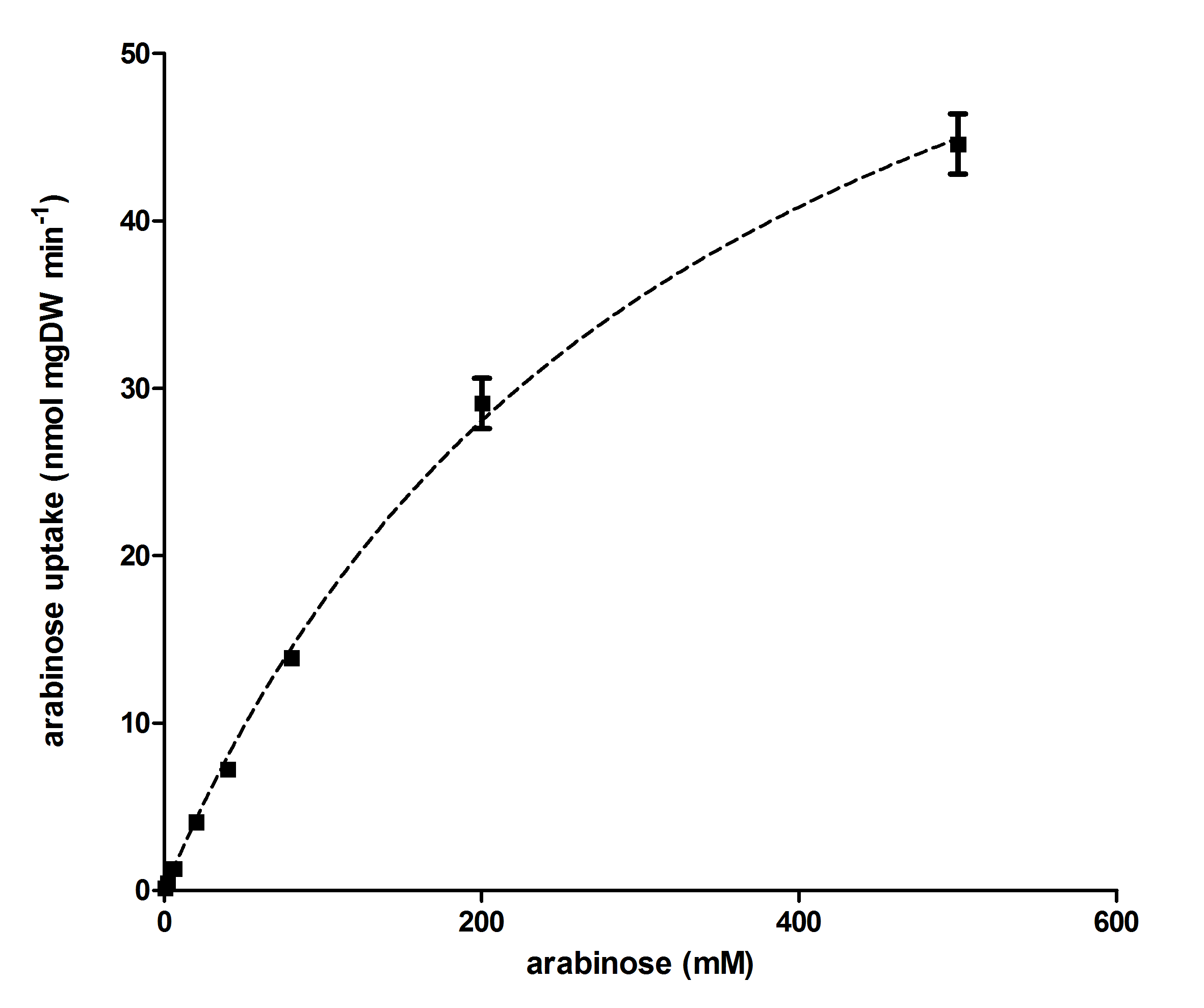

Supplement: Supplementary file 6 — Additional file 6. Specific rate of l-arabinose uptake by Gal2. Uptake experiments were performed with increasing concentrations of [14C-] l-arabinose with the Hxt1-7 and Gal2 deletion strain S. cerevisiae DS68625-GAL2 expressing GAL2 on a centromeric plasmid. Data are derived from duplicate experiments and shown as the average ± mean deviation. [file 13068_2018_1047_MOESM6_ESM.docx]

Additional File 7


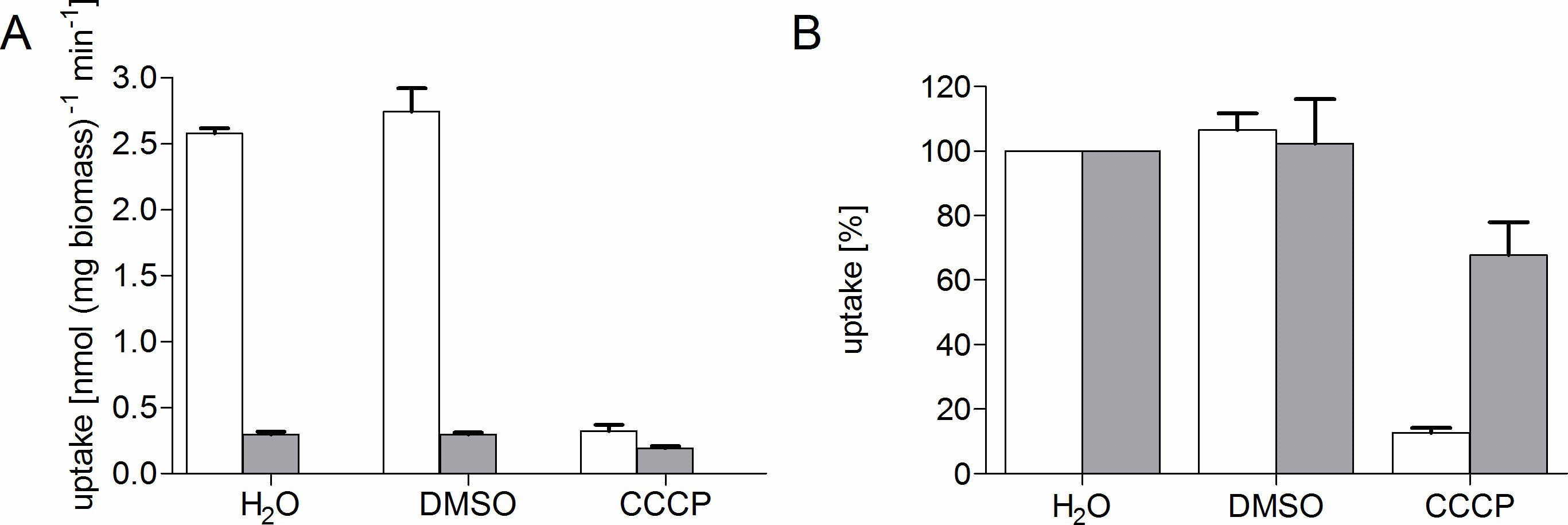

Supplement: Supplementary file 7 — Additional file 7. Impact of proton-gradient uncoupling on transport activity. Transport rates of [14C]-l-arabinose of the Hxt1-7 and Gal2 deletion strains DS68625-PcaraT and DS68625-GAL2 expressing either PcAraT (DS68625-PcaraT, white bars) or Gal2 (DS68625-GAL2, grey bars) on a centromeric plasmid. Transport rates were determined in 200 μl synthetic medium at a [14C]-l-arabinose concentration of 2 mmol L−1 upon addition of either 0.5 µL water, 0.5 μL DMSO, or 10 μM CCCP (0.5 µl of a stock solution dissolved in 100% DMSO) (A). Panel (B) shows the uptake capacity in % relative to the control (H2O). Data are derived from duplicate experiments and shown as the average ± mean deviation. [file 13068_2018_1047_MOESM7_ESM.docx]
